# Nuclear PD-L1 promotes EGR1-mediated angiogenesis and accelerates tumorigenesis

**DOI:** 10.1038/s41421-023-00521-7

**Published:** 2023-03-28

**Authors:** Jie Yu, Ai Zhuang, Xiang Gu, Yu Hua, Ludi Yang, Shengfang Ge, Jing Ruan, Peiwei Chai, Renbing Jia, Xianqun Fan

**Affiliations:** Department of Ophthalmology, Shanghai Key Laboratory of Orbital Diseases and Ocular Oncology, Shanghai Ninth People’s Hospital, Shanghai JiaoTong University School of Medicine, Shanghai, China

**Keywords:** Tumour angiogenesis, Cancer therapy

## Abstract

Targeting programmed cell death protein ligand 1 (PD-L1) remains one of the most essential immunotherapies in cancer^1,2^. PD-L1 has been detected in the nucleus in multiple malignancies, playing an oncogenic role independent of immune checkpoint regulation^3–5^. Howbeit, the regulatory function of nuclear PD-L1 (nPD-L1) remains to be fully understood. Here, we report that nPD-L1 is an endogenous accelerator for cancer angiogenesis. First, we found that an abundant proportion of PD-L1 was distributed within the nucleus of uveal melanoma samples, which is associated with an unfavorable outcome. Moreover, the capacity of promoting angiogenesis was largely attenuated in the nPD-L1-deficient cells both in vivo and in vitro. Mechanistically, nPD-L1 facilitates p-STAT3 binding to the promoter of *early growth response-1* (*EGR1*), resulting in the activation of EGR1-mediated angiogenesis. Therapeutically, the inhibition of histone deacetylase 2 restores the normal acetylation level of PD-L1, blocking its nuclear translocation and thereby attenuating tumor angiogenesis. Conclusively, we reveal that nPD-L1 promotes angiogenesis in malignancies, and provide a novel anti-vascularization strategy through blocking aberrant PD-L1 nuclear translocation for tumor therapy.

## Introduction

Programmed death-ligand 1 (PD-L1), expressed in multiple cancer cells as well as diversified immune cells, serves as one of the most essential immune-inhibitory molecules^6,7^. PD-L1 interacts with PD-1 on T cells and shuts down the immune response against host cells in order to avoid autoimmune diseases under physiological conditions^8,9^. However, in many cancers, upregulated PD-L1 on cancer cells hijacks this system and inhibits the activation of T cells in the tumor microenvironment, thus leading to immune escape during tumorigenesis^10^. The selective inhibition of the PD1/PD-L1 interaction is expected to maintain the host defense system and contribute to cancer eradication^11^, including urothelial bladder cancer^12^, non-small-cell lung cancer^13^, hepatocellular carcinoma^14^, skin cutaneous melanoma^15,16^, and mucosal melanoma^17,18^.

Despite the success of PD1/PD-L1 inhibitors in skin cutaneous melanoma as well as mucosal melanoma, the response of anti-PD1/PD-L1 is rather limited in uveal melanoma (UM), the most frequent and deadly intraocular malignancy in adults^17,19^. Although most UM cases present positive PD-L1 signals in tumors^20,21^, numerous clinical studies have demonstrated that UM patients are resistant to anti-PD1/PD-L1 therapies^22–25^. The lack of an in-depth understanding of the resistance of anti-PD1/PD-L1 unavoidably hinders the development of the immunotherapy in UM patients.

Intriguingly, PD-L1 is a multifunctional molecule that can be translocated into the nucleus, conferring resistance towards common anti-PD1/PD-L1 therapies^3^. nPD-L1 plays an oncogenic role independent of immune checkpoint regulation. For example, nPD-L1 promoted triple-negative breast cancer growth by controlling proper sister chromatid cohesion and segregation^4^. In addition, histone deacetylase 2 (HDAC2)-dependent deacetylation of membrane PD-L1 promotes its nuclear translocation, regulating the immune response in breast cancer and colon cancer^3^. Furthermore, p-STAT3 facilitates nPD-L1 translocation, facilitating the apoptosis-to-pyroptosis switch under hypoxic stress in breast cancer^5^. These findings highlighted the oncogenic functions of nPD-L1; however, the role of nPD-L1 in angiogenesis regulation has rarely been reported.

Herein, we identified that a large proportion of PD-L1 signals were specifically distributed within the nucleus in UM samples rather than in other kinds of melanomas. An elevated proportion of nPD-L1 subsequently promoted angiogenesis and accelerated tumorigenesis, both in vitro and in vivo. Interestingly, nPD-L1 recruited p-STAT3 to the promoter of *early growth response-1* (*EGR1*) and activated EGR1-mediated vasculogenesis. Therapeutically, we revealed that HDAC2 inhibition blocked PD-L1 nuclear translocation and attenuated angiogenesis. Taken together, we reveal a novel oncogenic role of nPD-L1 and provide a therapeutic target for effective antiangiogenic treatment in cancers.

## Results

### Increased nPD-L1 intensity refers to an unfavorable prognosis in UM

**Fig. 1:**
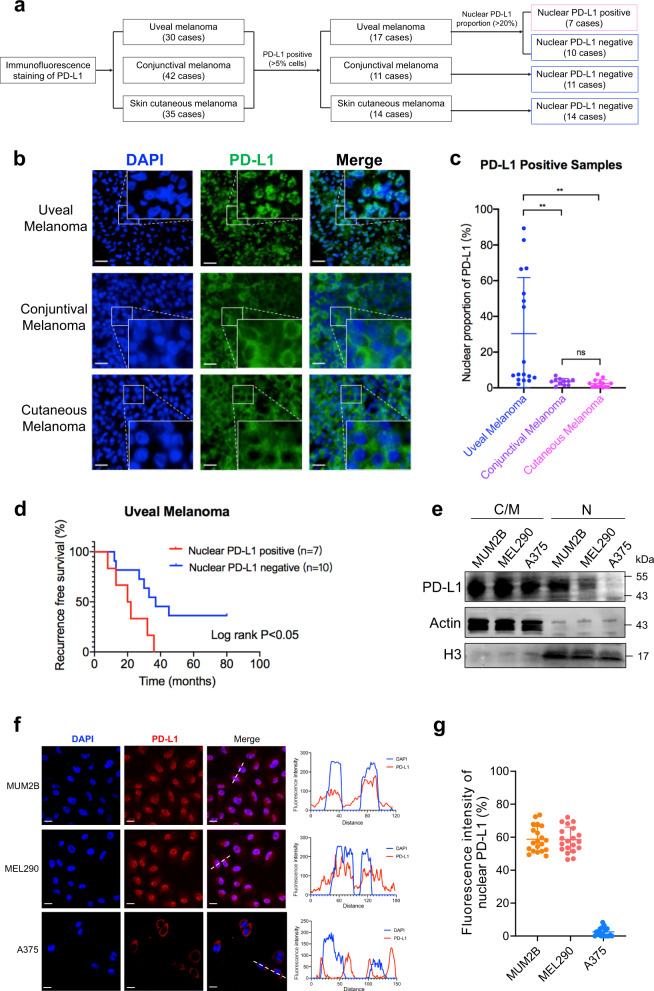
Elevated nuclear proportion of PD-L1 in UM. **a** Flow diagram for the identification of PD-L1 subcellular localization in different types of melanomas. **b** Representative images of IF staining of PD-L1 in UM, CoM and SKCM samples. Scale bars, 25 µm. **c** Statistical results of the nPD-L1 proportion in UM, CoM and SKCM samples. Data are presented as means ± SD. Two-tailed unpaired Student’s *t*-tests. **d** Kaplan–Meier analysis of the recurrence-free survival of nPD-L1-positive and -negative patients. A log-rank test was used to determine the statistical significance between the nPD-L1-positive group (*n* = 7) and the nPD-L1-negative group (*n* = 10). **e** Western blot analysis of cytoplasmic/membrane (C/M) and nuclear (N) fractions of PD-L1 in different types of melanoma cell lines. **f** IF staining of PD-L1 in different types of melanoma cell lines. Left panel, representative IF staining images. Scale bars, 10 µm. Right panel, colocalization analysis of PD-L1 and DAPI by ImageJ software. **g** Statistical analysis of nPD-L1 fluorescence intensity (%) in Fig. 1f. *n* = 20. Data are presented as the means ± SD. Two-tailed unpaired Student’s *t*-tests.

To investigate the subcellular localization of PD-L1 in different types of melanomas, we performed immunofluorescence (IF) staining in a cohort of 30 UM cases, 42 conjunctival melanoma (CoM) cases and 35 skin cutaneous melanoma (SKCM) cases. Notably, we observed that 57% (17/30) of UM, 26% (11/42) of CoM and 40% (14/35) of SKCM samples presented a positive PD-L1 signal (> 5% cells expressing PD-L1, PD-L1^+^) (Fig. 1a). Intriguingly, almost all PD-L1 was localized at the membrane in CoM and SKCM, which is consistent with the favorable response towards anti-PD1/PD-L1 therapy in these tumors. However, for PD-L1^+^ UM, a large proportion of PD-L1 was identified within the nucleus in 7 cases (Fig. 1b, c). Importantly, elevated nPD-L1 intensity indicated earlier tumor recurrence (log-rank *P* < 0.05; Fig. 1d; Supplementary Fig. S1a). Interestingly, the Cancer Genome Atlas (TCGA) database showed that higher PD-L1 level was correlated with worse prognosis in UM, but a favorable outcome for SKCM (Supplementary Fig. S1b, c), which is in perfect alignment with our observation that PD-L1 was distributed differently between these two kinds of melanomas.

Moreover, we tested the PD-L1 expression across different melanoma cell lines. We found that two UM (MUM2B and MEL290) and three SKCM (A375, M21 and LOX-IMVI) cell lines presented positive PD-L1 signals (Supplementary Fig. S1d, e). Consistently, PD-L1 was abundantly distributed in the nucleus of UM cells but localized exclusively in the membrane of SKCM cells, as measured by western blot detection of cytoplasmic/membrane (C/M) and nuclear (N) fractions (Fig. 1e; Supplementary Fig. S1f) and IF assay (Fig. 1f, g). Collectively, these data demonstrated that nPD-L1 was significantly elevated in UM samples and cell lines, but not in other kinds of melanomas.

### nPD-L1 facilitated angiogenesis in UM

**Fig. 2:**
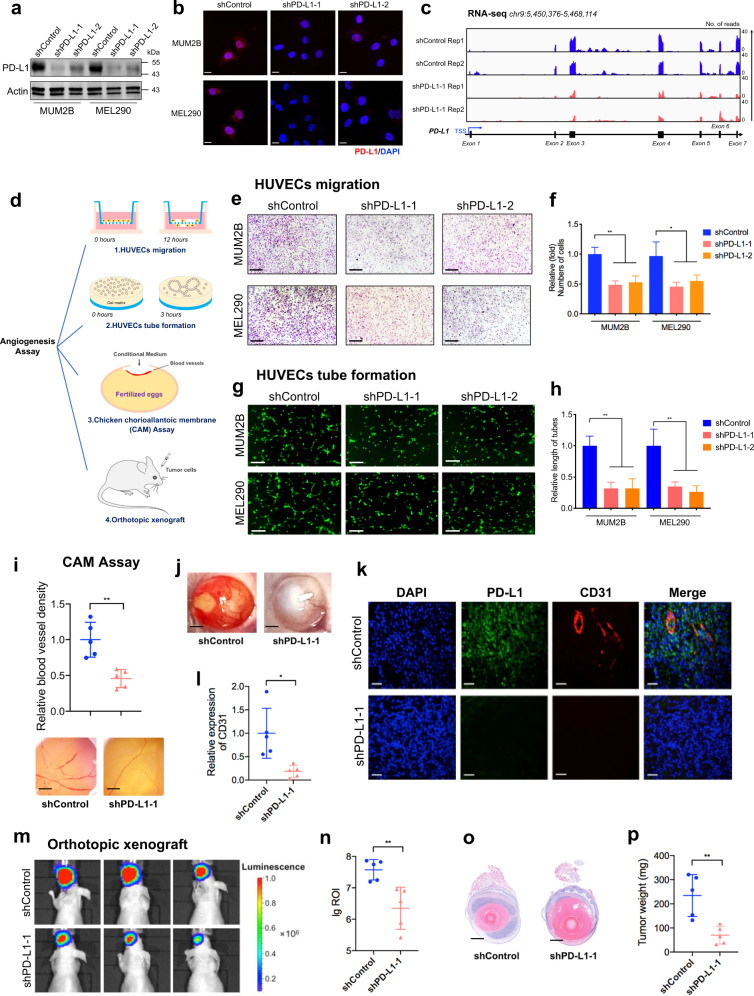
PD-L1 facilitates angiogenesis in UM. **a**, **b** Stable silencing of PD-L1 in the UM cell lines MUM2B and MEL290 verified by western blot (**a**) and IF staining (**b**). **c** IGV tracks for PD-L1 expression from RNA-seq analysis of PD-L1-silenced cells and control cells. **d** Schematic diagram summarizing the angiogenesis assays used to determine the role of PD-L1, including HUVEC migration, HUVEC tube formation, CAM assays, and orthotopic xenografts. **e** HUVEC migration assay that investigates the effect of conditional medium from PD-L1-silenced cells and control cells on the migration ability of HUVECs. Scale bars, 100 µm. **f** Statistical analysis of the HUVEC migration assay. *n* = 3. Data are presented as means ± SD. Two-tailed unpaired Student’s *t*-test. **g** HUVEC tube formation assay that investigates the effect of conditional medium from PD-L1-silenced cells and control cells on HUVEC tubule formation. Scale bars, 50 µm. **h** Statistical analysis of the HUVEC tube formation assay. *n* = 3. Data are presented as means ± SD. Two-tailed unpaired Student’s *t*-test. **i** Representative images and quantitative analysis of blood vessels of CAM treated with conditional medium from PD-L1-silenced cells and control cells. *n* = 5. Data are presented as means ± SD. Two-tailed unpaired Student’s *t*-test. **j** Representative images of UM orthotopic xenografts generated by MUM2B cells expressing control or PD-L1 shRNA. **k** IF images of PD-L1 and CD31 in UM orthotopic xenografts. Scale bars, 40 µm. **l** Statistical analysis of CD31 expression in UM orthotopic xenografts. *n* = 5. Data are presented as means ± SD. Two-tailed unpaired Student’s *t*-test. **m** Representative bioluminescent images of UM orthotopic xenografts. **n** Statistical analysis of the bioluminescent signals in UM orthotopic xenografts. *n* = 5. Data are presented as means ± SD. Two-tailed unpaired Student’s *t*-test. **o** Representative images of H&E staining of UM orthotopic xenografts. **p** Statistical analysis of the tumor weight. *n* = 5. Data are presented as means ± SD. Two-tailed unpaired Student’s *t*-test.

To determine the function of nPD-L1 during the pathogenesis of UM, we generated two individual shRNAs for silencing PD-L1. We found that PD-L1 expression was significantly diminished after shRNA transfection, as demonstrated by quantitative real-time PCR (qRT-PCR, Supplementary Fig. S2a), western blot (Fig. 2a), IF staining (Fig. 2b) and RNA-seq (deposited in GEO database: GSE202884; Fig. 2c) assays. Importantly, PD-L1 silencing did not alter proliferation (Supplementary Fig. S2b), colony formation ability (Supplementary Fig. S2c, d) or cell migration capacity (Supplementary Fig. S2e, f) in all tested UM cell lines.

Given that the choroid membrane is an intraocular blood vessel formed by endothelial cells (ECs) and that the “angiogenic switch” facilitates malignant transformation, serving as an important parameter in UM^26,27^, we then tested the role of nPD-L1 in tumor–ECs interactions during the tumorigenesis of UM (Fig. 2d). Strikingly, the human umbilical vein endothelial cell line (HUVEC) showed decreased migration and tube formation after co-cultured with conditional medium derived from PD-L1-silenced UM cells (Fig. 2e–h). Furthermore, we performed a chicken chorioallantoic membrane (CAM) assay and generated orthotopic xenografts with luciferase-labeled PD-L1 knockdown UM cells to evaluate the role of PD-L1 in angiogenesis in vivo. The PD-L1-silenced UM cells showed decreased pro-angiogenic capacity in CAM assay (Fig. 2i). Interestingly, we observed that most orthotopic xenografts were surrounded by affluent blood vessels in the control group (Fig. 2j, left); however, almost no visible blood vessels were observed in PD-L1-silenced UM group (Fig. 2j, right). Consistently, we found a decreased signal of CD31, a marker of angiogenesis^28^, in PD-L1-silenced xenografts (Fig. 2k, l). Moreover, animal bioluminescence imaging demonstrated a diminished tumor signal in the PD-L1-silenced group, which is consistent with the attenuated angiogenesis capacity after silencing PD-L1 (Fig. 2m, n). The tumor size and weight of the PD-L1-silenced group were also significantly lower than those of the control group (Fig. 2o, p). Taken together, these data indicated that nPD-L1 could promote angiogenesis in UM, both in vitro and in vivo.

Soluble PD-L1 has been reported to contribute to immune-escape, which is associated with an advanced stage of malignant melanoma (Stage IV)^29^. We then added recombinant PD-L1 into PD-L1-depleted UM cells at a dose of 10 µg/mL for 72 h, as previous decribed^29^. However, HUVEC migration (Supplementary Fig. S3a, b) and tube formation (Supplementary Fig. S3c, d) assays showed that recombinant PD-L1 failed to restore the pro-angiogenic function of PD-L1-depleted cells. Since secreted PD-L1 is not distributed within the nucleus, this observation further indicates that nuclear PD-L1 harbors the pro-angiogenic function, rather than its soluble form.

We further adopted two individual sgRNAs to completely delete endogenous PD-L1 (Supplementary Fig. S4a). In line with PD-L1-shRNAs-treated cells, PD-L1 knockout also did not alter proliferation (Supplementary Fig. S4b), colony formation ability (Supplementary Fig. S4c, d) or cell migration capacity (Supplementary Fig. S4e, f). In addition, PD-L1-knockout cells presented a decreased capacity of promoting HUVEC migration (Supplementary Fig. S4g, h) and tube formation (Fig. S4i, j) compared with control cells. Consistently, these data further indicated that nuclear PD-L1 facilitates angiogenesis in UM, rather than regulates the capacities of proliferation, colony formation and migration of cancer cells.

### nPD-L1 transcriptionally activated *EGR1* expression

**Fig. 3:**
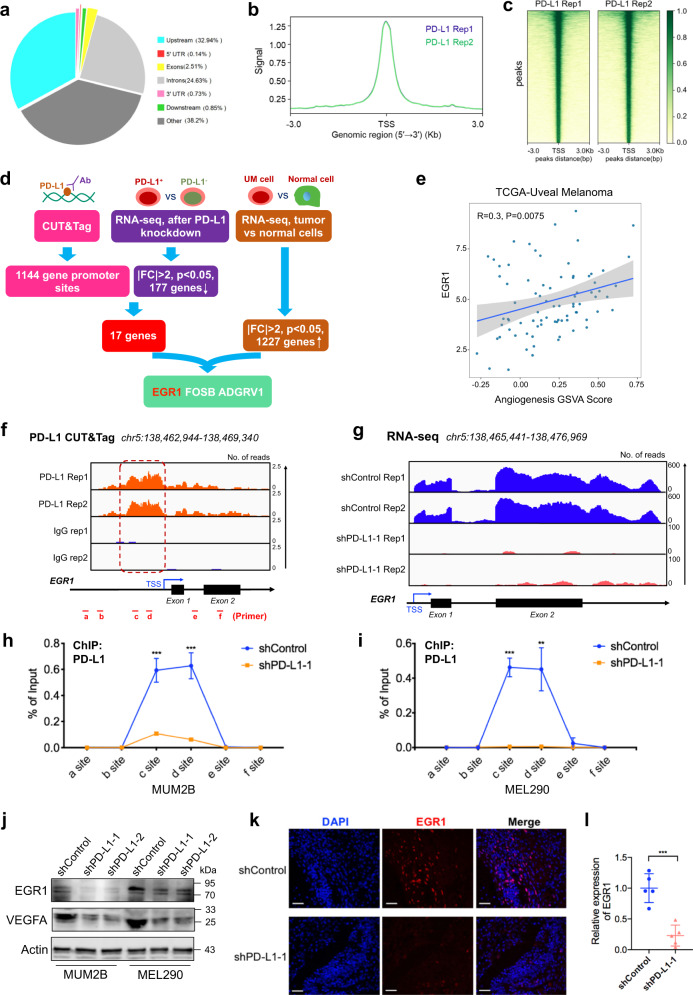
Nuclear PD-L1 activates EGR1 transcription. **a** The genomic distribution of PD-L1 CUT&Tag peaks in MUM2B cells. **b** PD-L1 CUT&Tag signal height and position relative to transcription start sites (TSSs) for all genes in MUM2B cells. Two replicates are shown. **c** CUT&Tag density heatmap of PD-L1 enrichment in MUM2B cells within 3 kb around TSS. **d** Bioinformatics analysis identified *EGR1* as a downstream target of nPD-L1. **e** Correlation plot of EGR1 expression and angiogenesis GSVA score in the TCGA-UM cohort. Correlation coefficients were calculated by the Spearman test. **f** IGV tracks for *EGR1* from PD-L1 CUT&Tag analysis. **g** IGV tracks for *EGR1* expression from RNA-seq analysis of PD-L1-silenced cells and control cells. **h**, **i** ChIP-qPCR assay of the PD-L1 status at the *EGR1* promoter and its upstream and downstream regions after PD-L1 silencing in MUM2B (**h**) and MEL290 (**i**) cells. **j** Western blot images showing the protein levels of EGR1 and VEGFA in PD-L1-silenced cells and control cells. **k** IF images of EGR1 in UM orthotopic xenografts. Scale bars, 50 µm. **l** Statistical analysis of EGR1 expression in UM orthotopic xenografts. *n* = 5. Data are presented as means ± SD. Two-tailed unpaired Student’s *t*-test.

To unveil the mechanism underlying the pro-angiogenic function of nPD-L1, we first performed RNA-seq after silencing PD-L1 and observed a dramatic change in the transcriptional pattern, with 177 downregulated genes and 73 upregulated genes (Supplementary Fig. S5a). Kyoto Encyclopedia of Genes and Genomes (KEGG) analysis revealed that these dysregulated genes were enriched in tumor-related pathways (Supplementary Fig. S5b). Furthermore, we performed CUT&Tag using anti-PD-L1 antibodies to visualize the nPD-L1 distribution in the genome. We identified 1144 nPD-L1-binding sites, which were mainly distributed near the transcription start site (TSS), suggesting a transcriptional regulation function of nPD-L1 (deposited in GEO database: GSE202394, Fig. 3a–c). Notably, combining the CUT&Tag and RNA-seq data, 17 genes presented PD-L1-binding signals in their promoters and decreased mRNA expressions after silencing PD-L1. Moreover, of these 17 genes, only 3 genes demonstrated increased expression (|FC| > 2, *P* < 0.05) in tumors compared to normal control cells, including *EGR1* (Fig. 3d), a positive regulator of angiogenic factors^30,31^. As expected, in UM, EGR1 showed a strong positive correlation with an elevated GSVA score of the angiogenic signaling pathway (*R* = 0.30, *P* = 0.0075) in the TCGA cohort, suggesting that EGR1 functions as a pro-angiogenic regulator in UM (Fig. 3e).

We then focused on the regulatory role of nPD-L1 in the *EGR1* locus. Well-defined PD-L1 peaks were observed in the *EGR1* promoter in the CUT&Tag data, indicating that nPD-L1 is located in the *EGR1* promoter (Fig. 3f). In addition, the chromatogram of RNA-seq data indicated attenuated *EGR1* expression in PD-L1-silenced cells (Fig. 3g). Consistent with these high-throughput results, ChIP-qPCR assays further confirmed the binding of PD-L1 to the *EGR1* promoter, and the binding level was decreased in PD-L1-silenced cells (Fig. 3h, i). It was reported that EGR1 potentially promotes VEGFA expression for angiogenesis^32^. Consistently, the expressions of EGR1 and VEGFA were significantly decreased in PD-L1-silenced cells at both the mRNA (Supplementary Fig. S5c–e) and protein (Fig. 3j) levels. As expected, orthotopic xenograft tumors derived from the PD-L1-silenced cells also displayed limited EGR1 expression (Fig. 3k, l). Overall, these findings demonstrated that EGR1 could be transcriptionally activated by nPD-L1.

### The promotion of angiogenesis by nPD-L1 depends on EGR1 activation

Although EGR1 is a canonical pro-angiogenic regulator^30,31^, the function of EGR1 in UM remains unknown. Therefore, we first knocked down EGR1 by shRNAs in UM cells, and observed significant reductions in VEGFA expression at both the mRNA (Supplementary Fig. S6a, b) and protein levels (Supplementary Fig. S6c). Moreover, conditional medium derived from EGR1-deficient cells was unable to accelerate migration and tube formation abilities of endothelial cells; however, a significant promotion was noted in the control group (Supplementary Fig. S6d–g). Taken together, these results support the fact that EGR1 serves as an important accelerator for angiogenesis in UM.

**Fig. 4:**
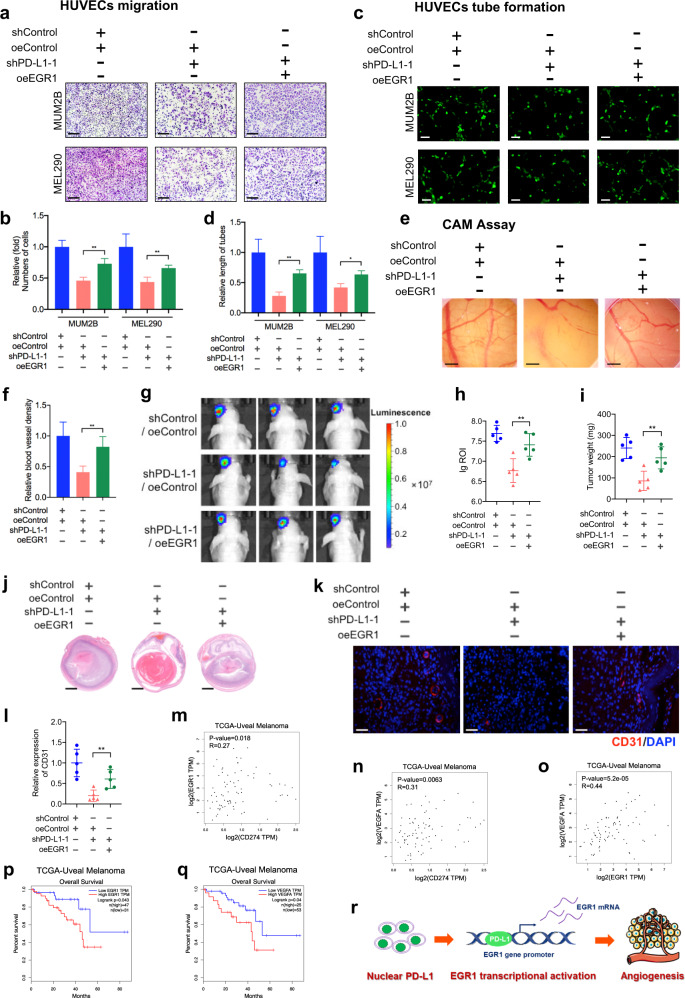
Nuclear PD-L1 facilitates angiogenesis through EGR1. **a** HUVEC migration assay that investigates the effect of EGR1 re-expression in PD-L1-silenced cells on the migration ability of HUVECs. Scale bars, 100 µm. **b** Statistical analysis of the HUVEC migration assay. *n* = 3. Data are presented as means ± SD. Two-tailed unpaired Student’s *t*-test. **c** HUVEC tube formation assay that investigates the effect of EGR1 re-expression in PD-L1-silenced cells on HUVEC tubule formation. Scale bars, 25 µm. **d** Statistical analysis of the HUVEC tube formation assay. *n* = 3. Data are presented as means ± SD. Two-tailed unpaired Student’s *t*-test. **e** CAM assay that investigates the effect of EGR1 re-expression in PD-L1-silenced cells on the blood vessel density of CAM. **f** Statistical analysis of the CAM assay. *n* = 5. Data are presented as means ± SD. Two-tailed unpaired Student’s *t*-test. **g** Representative bioluminescent images of UM orthotopic xenografts generated by PD-L1-silenced cells that re-express EGR1. **h** Statistical analysis of the bioluminescent signals in UM orthotopic xenografts. *n* = 5. Data are presented as means ± SD. Two-tailed unpaired Student’s *t*-test. **i** Statistical analysis of the tumor weight. *n* = 5. Data are presented as means ± SD. Two-tailed unpaired Student’s *t*-test. **j** Representative images of H&E staining of UM orthotopic xenografts generated by PD-L1-silenced cells that re-express EGR1. **k** IF images of CD31 in UM orthotopic xenografts constructed by EGR1 re-expression in PD-L1-silenced cells. Scale bars, 40 µm. **l** Statistical analysis of CD31 expression in UM orthotopic xenografts. *n* = 5. Data are presented as means ± SD. Two-tailed unpaired Student’s *t*-test. **m**–**o** Gene expression correlation analysis of *PD-L1* and *EGR1* (**m**), *PD-L1* and *VEGFA* (**n**), *EGR1* and *VEGFA* (**o**) in UM patients using TCGA database. **p**, **q** Kaplan–Meier analysis of the overall survival of UM patients with low and high EGR1 (**p**) and VEGFA (**q**) expression levels using TCGA database. **r** Schematic diagram showing that nPD-L1 facilitates angiogenesis in UM through transcriptional activation of *EGR1*.

To further verify that EGR1 was responsible for nPD-L1-mediated pro-angiogenesis, we reintroduced EGR1 into PD-L1-silenced cells (Supplementary Fig. S7a). Obviously, HUVEC migration (Fig. 4a, b), tube formation (Fig. 4c, d) and CAM assays (Fig. 4e, f) showed that restoration of EGR1 largely rescued the pro-angiogenesis capacity of PD-L1-silenced cells. Next, in the orthotopic xenografts assay, we observed that the restoration of EGR1 rescued the inhibited tumor growth (Fig. 4g–j) and angiogenesis (Fig. 4k, l) in PD-L1-silenced tumors, unambiguously demonstrating that EGR1 functions as a necessary regulator of the pro-angiogenic function of nPD-L1. In addition, we reintroduced VEGFA into PD-L1-silenced cells (Supplementary Fig. S8a). Consistently, HUVEC migration (Supplementary Fig. S8b, c) and tube formation (Supplementary Fig. S8d, e) assays showed that restoration of VEGFA largely rescued the pro-angiogenesis capacity of PD-L1-silenced cells, which is in alignment with ectopic expression of EGR1. Thus, these data further indicate that nPD-L1 facilitates angiogenesis through activating EGR1/VEGFA signaling.

Since anti-VEGF therapy is one of the most important treatments for multiple cancers^33–36^, we further detected the therapeutic efficiency of anti-VEGF therapy in UM. As expected, when used with a dose of 5 mg/kg twice per week, anti-VEGF therapy significantly inhibited tumor growth (Supplementary Fig. S9a–d). Notably, in PD-L1^–^ UM cells, although anti-VEGF still triggered a significant reduction of tumor growth in vivo, the efficacy was largely compromised when compares with control group (Supplementary Fig. S9a–d). This result agrees with our observation that EGR1/VEGFA serves as downstream factors of nPD-L1 and further underscores that pro-angiogenic function is necessary in the nPD-L1-mediated tumor growth.

Importantly, in the TCGA UM cohort, we identified positive expression correlations between *PD-L1* and *EGR1* (*R* = 0.27, *P* = 0.018), *PD-L1* and *VEGFA* (*R* = 0.31, *P* = 0.0063), and *EGR1* and *VEGFA* (*R* = 0.44, *P* = 5.2e–05) in UM (Fig. 4m–o). Consistent with PD-L1, higher EGR1 or VEGFA expression levels correlated with an unfavorable prognosis in UM (Fig. 4p, q; Supplementary Fig. S7b, c). Therefore, these data indicated that nPD-L1 facilitates angiogenesis in an EGR1-dependent manner during UM progression (Fig. 4r).

As A375 cells specifically presented salient membrane signals of PD-L1, we then explored the role of membrane PD-L1 in angiogenesis by using A375 cells as a control. In contrast, we did not find that PD-L1 silencing led to a significant reduction in EGR1 or VEGFA expression in A375 cells (Supplementary Fig. S10a–c). Moreover, PD-L1 silencing did not attenuate HUVEC migration and tube formation capacities (Supplementary Fig. S10d–g). Moreover, no significant correlation between *PD-L1* and *EGR1* or *PD-L1* and *VEGFA* was observed in the TCGA-SKCM cohort (Supplementary Fig. S10h, i). Taken together, these results demonstrated that membrane PD-L1 failed to facilitate angiogenesis.

### nPD-L1 enhanced p-STAT3 binding to the *EGR1* promoter

**Fig. 5:**
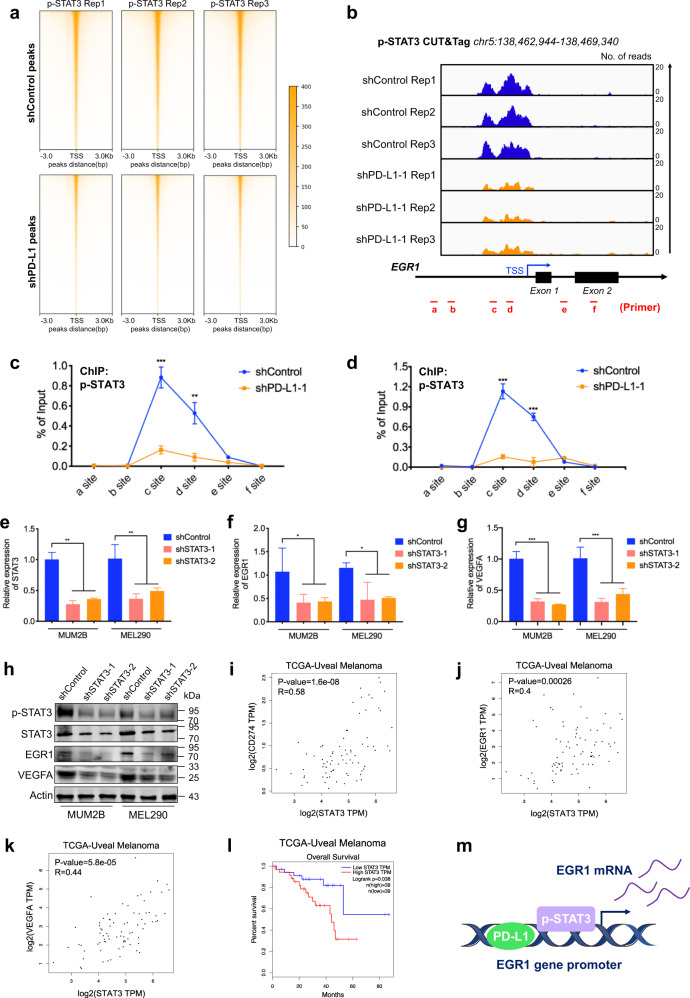
Nuclear PD-L1 promotes transcriptional activation of EGR1 by p-STAT3. **a** CUT&Tag density heatmap of p-STAT3 enrichment in PD-L1-silenced cells and control cells within 3 kb around TSS. **b** IGV tracks for *EGR1* from p-STAT3 CUT&Tag analysis of PD-L1-silenced cells and control cells. **c**, **d** ChIP–qPCR assay of the p-STAT3 status at the *EGR1* promoter and its upstream and downstream regions after PD-L1 silencing in MUM2B (**c**) and MEL290 (**d**) cells. **e**–**g** RT-PCR detection of relative *STAT3* (**e**), *EGR1* (**f**) and *VEGFA* (**g**) mRNA expression in MUM2B and MEL290 cells after STAT3 knockdown. *n* = 3. Data are presented as means ± SD. Two-tailed unpaired Student’s *t*-test. **h** Western blot images showing the protein levels of p-STAT3, STAT3, EGR1 and VEGFA in STAT3-silenced cells and control cells. **i**–**k** Gene expression correlation analysis of *STAT3* and *PD-L1* (**i**), *STAT3* and *EGR1* (**j**), *STAT3* and *VEGFA* (**k**) in UM patients using TCGA database. **l** Kaplan-Meier analysis of the overall survival of UM patients with low and high STAT3 expression levels using TCGA database. **m** Schematic diagram showing that nPD-L1 and p-STAT3 coactivated *EGR1* transcription in UM.

Given that phosphorylated STAT3 (p-STAT3) and nPD-L1 act as coactivators in transcription^5^, we then explored whether p-STAT3 participates in nPD-L1-mediated transcriptional activation of *EGR1*. First, as demonstrated by p-STAT3 CUT&Tag data, we observed that the genome-wide binding intensity of p-STAT3 was compromised in PD-L1-deficient cells (deposited in GEO database: GSE202883; Fig. 5a; Supplementary Fig. S11a), which agrees with the previous observation that p-STAT3 serves as the co-activator of nPD-L1 in transcriptional activation^5^. Notably, the change in the binding loci of p-STAT3 was associated with multiple oncogenic pathways, indicating that PD-L1-mediated p-STAT3 regulation plays a vital role in regulating UM (Supplementary Fig. S11b).

Most importantly, we identified an evident enrichment of p-STAT3 in the *EGR1* promoter in UM, while attenuated signals were observed after knocking down PD-L1 (Fig. 5b). Consistently, as validated by chromatin immunoprecipitation, we observed that the binding intensity of p-STAT3 to the *EGR1* promoter was reduced in PD-L1-silenced cells (Fig. 5c, d), demonstrating that p-STAT3 binding requires nPD-L1 locating at the promoter of *EGR1*. Furthermore, we silenced STAT3 by transfecting two individual shRNAs, and STAT3 showed reduced expression at both mRNA (Fig. 5e) and protein (Fig. 5h, panel 1) levels. Notably, STAT3 silencing led to a dramatic decrease in both *EGR1* and *VEGFA* mRNA expressions (Fig. 5f–h). Accordingly, STAT3-deficient cells presented a compromised capacity of promoting HUVEC migration (Supplementary Fig. S12a, b) and tube formation (Supplementary Fig. S12c, d) compared with control cells. In addition, in the TCGA cohort, the *STAT3* expression level was positively correlated with *CD274* (*R* = 0.58, *P* = 1.6e–08), *EGR1* (*R* = 0.4, *P* = 0.00026), and *VEGFA* (*R* = 0.44, *P* = 5.8e–05) expression in clinical UM samples (Fig. 5i–k), which supported the conclusion that STAT3 mediated transcriptional activation of *EGR1*. Moreover, elevated STAT3 expression levels were associated with worse prognosis in UM patients (Fig. 5l). Conclusively, our results revealed that nPD-L1 enhanced p-STAT3 binding to the promoter of *EGR1*, thereby promoting *EGR1* expression (Fig. 5m).

### HDAC2-mediated PD-L1 deacetylation promoted its nuclear translocation

**Fig. 6:**
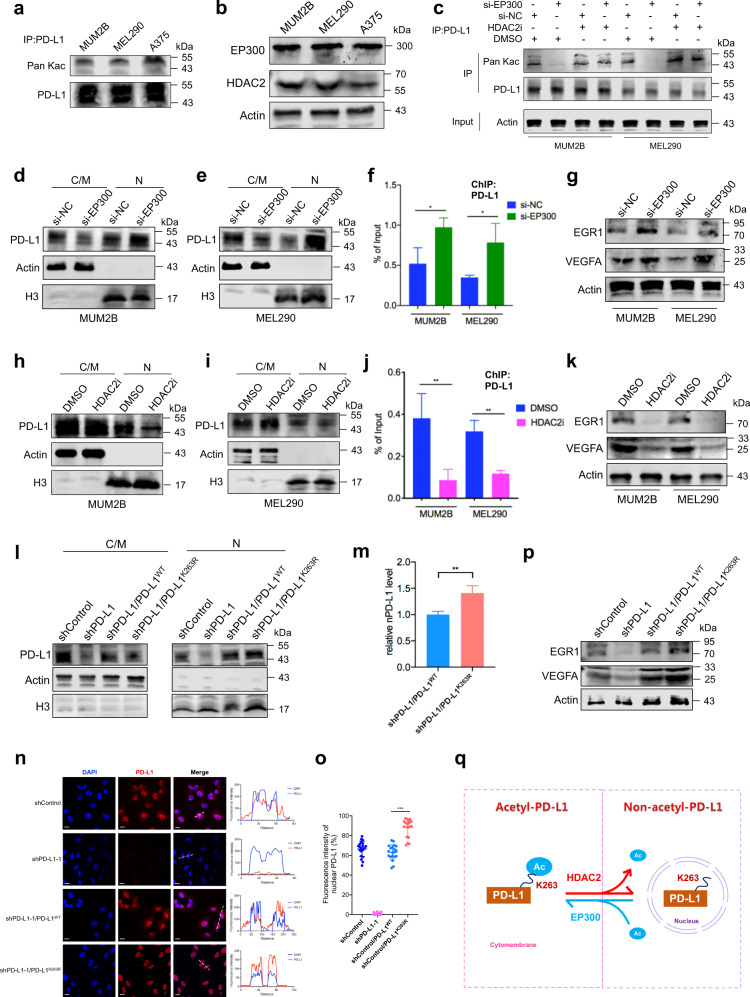
PD-L1 K263 acetylation abrogates its unclear translocation. **a** Western blot analysis of anti-PD-L1 IPs derived from MUM2B, MEL290 and A375 cells to detect PD-L1 acetylation levels. **b** Western blot images showing the protein levels of EP300 and HDAC2 in MUM2B, MEL290 and A375 cells. **c** Western blot analysis of anti-PD-L1 IPs derived from MUM2B and MEL290 cells treated with the indicated constructs to detect PD-L1 acetylation levels. **d**, **e** Western blot analysis of cytoplasmic/membrane (C/M) and nuclear (N) fractions of PD-L1 in MUM2B (**d**) and MEL290 (**e**) cells transduced with si-EP300. **f** ChIP-qPCR assay of the PD-L1 status at the *EGR1* promoter in MUM2B and MEL290 cells transduced with si-EP300. **g** Western blot images showing the protein levels of EGR1 and VEGFA in MUM2B and MEL290 cells transduced with si-EP300. **h**, **i** Western blot analysis of cytoplasmic/membrane (C/M) and nuclear (N) fractions of PD-L1 in MUM2B (**h**) and MEL290 (**i**) cells treated with 50 μM HDAC2i (santacruzamate A) for 24 h. **j** ChIP-qPCR assay of the PD-L1 status at the *EGR1* promoter in MUM2B and MEL290 cells treated with 50 μM HDAC2i for 24 h. **k** Western blot images showing the protein levels of EGR1 and VEGFA in MUM2B and MEL290 cells treated with 50 μM HDAC2i for 24 h. **l** Western blot analysis of cytoplasmic/membrane (C/M) and nuclear (N) fractions of PD-L1 in MUM2B cells treated with the indicated constructs. **m** Statistical analysis of nPD-L1 levels in MUM2B cells treated with the indicated constructs. *n* = 3. Data are presented as means ± SD. Two-tailed unpaired Student’s *t*-test. **n** IF staining of PD-L1 in MUM2B cells treated with the indicated constructs. Left panel, representative IF staining images. Scale bars, 10 µm. Right panel, co-localization analysis of PD-L1 and DAPI by ImageJ software. **o** Statistical analysis of nPD-L1 fluorescence intensity (%) in Fig. 6n. *n* = 20. Data are presented as means ± SD. Two-tailed unpaired Student’s *t*-test. **p** Western blot images showing the protein levels of EGR1 and VEGFA in MUM2B cells treated with the indicated constructs. **q** Schematic diagram showing that PD-L1 nuclear translocation was mediated by HDAC2-dependent PD-L1 K263 deacetylation.

We next explored the mechanism underlying the nuclear translocation of PD-L1 in UM. Since HDAC2-mediated deacetylation of PD-L1 (at the Lys263 site) facilitated its nuclear translocation, which could be rescued by EP300-induced PD-L1 acetylation^3^, we then tested whether the aberrant acetylation level leads to the formation of nPD-L1. Notably, nuclear-PD-L1^+^ cells exhibited decreased PD-L1 acetylation levels as compared with nuclear-PD-L1^–^ cells (Fig. 6a). Consistently, we also observed that HDAC2 level was significantly increased in these nuclear-PD-L1^+^ cells, whereas EP300 expression remained unchanged (Fig. 6b). These data indicated that the PD-L1 acetylation level negatively correlates with its nuclear signal. Consistent with a previous study^3^, we found that EP300-deficient cells presented decreased PD-L1 acetylation levels, while cells presented elevated PD-L1 acetylation levels after treatment with an HDAC2 inhibitor (santacruzamate A) (Fig. 6c).

Notably, we observed that the silencing of EP300 promoted PD-L1 nuclear translocation (Fig. 6d, e) and thereby increased the binding of PD-L1 to the promoter of *EGR1* (Fig. 6f**)**. In agreement with these observations, EGR1 and VEGFA levels were increased in EP300-deficient cells (Fig. 6g; Supplementary Fig. S13a, b). In contrast, when cells were treated with HDAC2i, the nuclear proportion of PD-L1 was decreased (Fig. 6h, i), giving rise to abrogated binding of PD-L1 to the promoter of *EGR1* (Fig. 6j). Accordingly, EGR1 and VEGFA levels were decreased after treatment with HDAC2i (Fig. 6k; Supplementary Fig. S13c, d). Therapeutically, HDAC2 inhibition suppressed the pro-angiogenic potential of UM cells, both in terms of the migration and tube formation capacities of HUVECs (Supplementary Fig. S14a–d). In addition, positive expression correlations between *HDAC2* and *PD-L1* (*R* = 0.36, *P* = 0.0013), *HDAC2* and *EGR1* (*R* = 0.27, *P* = 0.015), and *HDAC2* and *VEGFA* (*R* = 0.51, *P* = 1.5e–06) in the TCGA-UM cohort were noted (Supplementary Fig. S15a–c). Conclusively, these data indicate that HDAC2-mediated deacetylation promotes nPD-L1 formation, and thus promotes angiogenesis in UM cells.

**Fig. 7:**
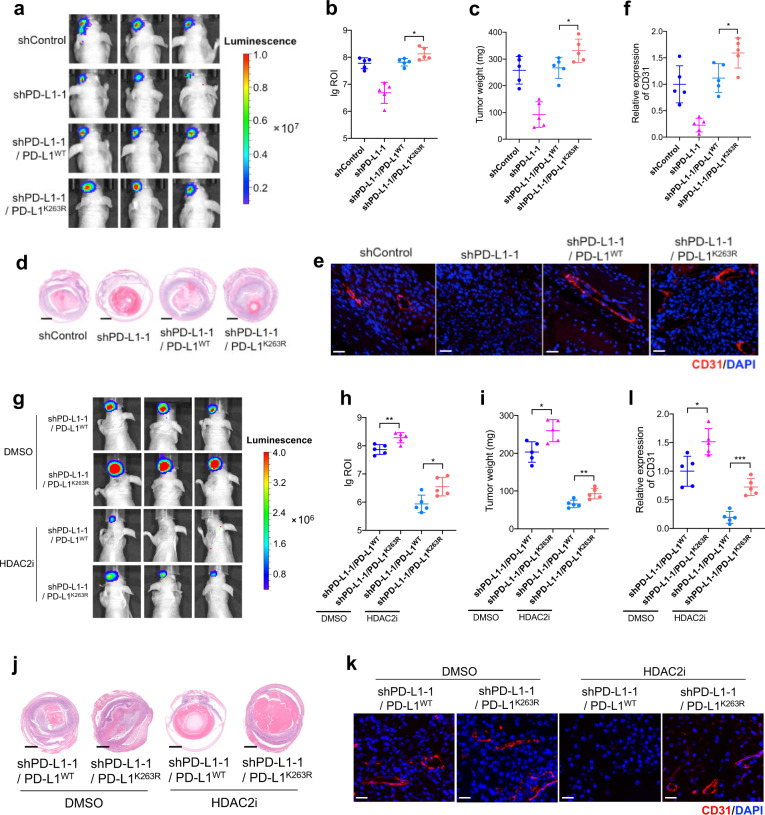
PD-L1 K263 deacetylation facilitates angiogenesis and tumorigenesis in vivo. **a** Representative bioluminescent images of UM orthotopic xenografts. **b** Statistical analysis of the bioluminescent signals in UM orthotopic xenografts. *n* = 5. **c** Statistical analysis of the tumor weight. *n* = 5. Data are presented as means ± SD. Two-tailed unpaired Student’s *t*-test. **d** Representative images of H&E staining of UM orthotopic xenografts generated by tumor cells with indicated constructs. **e** IF images of CD31 in UM orthotopic xenografts generated by tumor cells with indicated constructs. Scale bars, 40 µm. **f** Statistical analysis of CD31 expression in UM orthotopic xenografts. *n* = 5. Data are presented as means ± SD. Two-tailed unpaired Student’s *t*-test. **g** Representative bioluminescent images of UM orthotopic xenografts generated by tumor cells with indicated constructs and treated with HDAC2i. **h** Statistical analysis of the bioluminescent signals in UM orthotopic xenografts. *n* = 5. **i** Statistical analysis of the tumor weight. *n* = 5. Data are presented as means ± SD. Two-tailed unpaired Student’s *t*-test. **j** Representative images of H&E staining of UM orthotopic xenografts generated by tumor cells with indicated constructs and treated with HDAC2i. **k** IF images of CD31 in UM orthotopic xenografts generated by tumor cells with indicated constructs and treated with HDAC2i. Scale bars, 40 µm. **l** Statistical analysis of CD31 expression in UM orthotopic xenografts. *n* = 5. Data are presented as means ± SD. Two-tailed unpaired Student’s *t-*test.

To further validate the role of PD-L1 acetylation in its nuclear translocation, we reintroduced wild-type PD-L1 (PD-L1^WT^) and acetylation-deficient mutant PD-L1^K263R^ into PD-L1-silenced cells^3^. Notably, PD-L1^K263R^ showed an elevated nuclear proportion compared with PD-L1^WT^ (Fig. 6l–o). Moreover, EGR1 and VEGFA levels were significantly increased after reintroducing PD-L1^K263R^, rather than the exogenous PD-L1^WT^ (Fig. 6p; Supplementary Fig. S13e, f). In addition, the orthotopic xenograft assay showed that PD-L1^K263R^ exhibited increased capacity of promoting tumor growth (Fig. 7a–d) and angiogenesis (Fig. 7e, f) compared with PD-L1^WT^. Together, these data suggested that dynamic PD-L1 acetylation, serves as an important regulator in angiogenesis during carcinogenesis (Fig. 6q). To further confirm the role of acetylation-mimicking mutation of PD-L1 (K263Q) in PD-L1 localization and angiogenesis, we reintroduced PD-L1^WT^ and PD-L1^K263Q^ into PD-L1-knockout cells. However, no significant changes in nuclear proportion of PD-L1^K263Q^ were observed compared with PD-L1^WT^, as demonstrated by western blot detecting protein levels in nuclear/cytoplasmic fractions (Supplementary Fig. S16a) and IF assay (Supplementary Fig. S16b, c). Importantly, EGR1 and VEGFA levels remained unchanged after reintroducing PD-L1^K263Q^ (Supplementary Fig. S16d). These data indicate that the effect of K263Q is rather context-dependent and this mutant could not mimic a hyper-acetylated PD-L1 in our experiment system.

Since HDAC2 removes the acetylation modification of PD-L1 and promotes its nuclear transport, we then tested the response of wild-type PD-L1 and its acetylation-deficient mutant (PD-L1^K263R^) to HDAC2 inhibition. Compared to PD-L1^WT^ group, PD-L1^K263R^ is more resistant towards HDAC2 inhibition, with increased capacity of promoting tumor growth (Fig. 7g–j) and blood vessels formation (Fig. 7k, l). This results further proves the vital role of HDAC2-mediated-Lys263 deacetylation in the nPD-L1-mediated angiogenesis.

### Nuclear PD-L1 enhanced EGR1-mediated angiogenesis in breast cancer

**Fig. 8:**
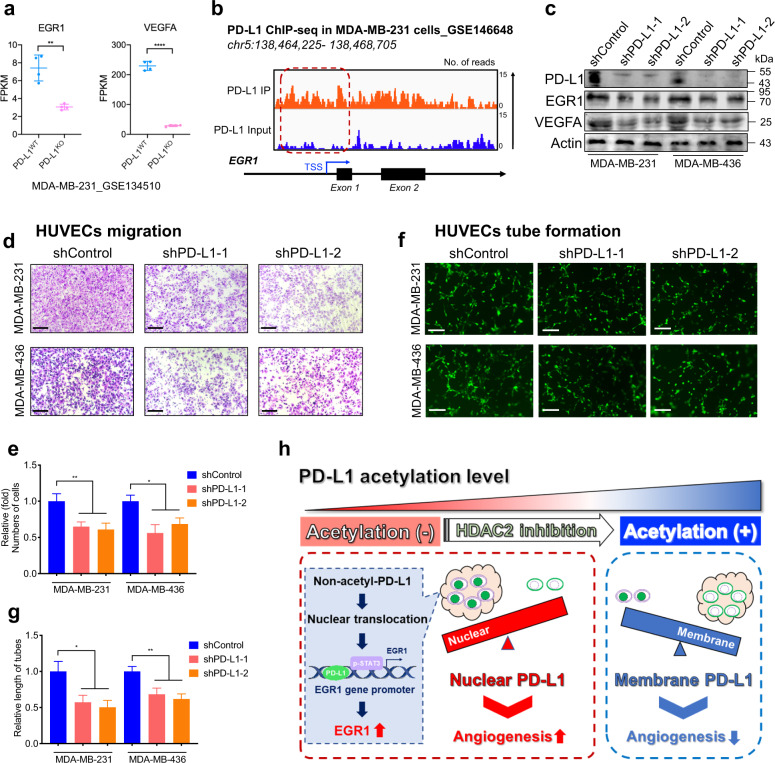
Nuclear PD-L1 enhanced EGR1 expression and angiogenesis in breast cancer. **a** FPKM levels of *EGR1* and *VEGFA* from RNA-seq analysis in breast cancer cell line MDA-MB-231 after PD-L1 knockout (GSE134510). *n* = 4. Data are presented as means ± SD. Two-tailed unpaired Student’s *t*-test. **b** IGV tracks for *EGR1* from PD-L1 ChIP-seq analysis in breast cancer cell line MDA-MB-231 (GSE146648). **c** Western blot images showing the protein levels of PD-L1, EGR1 and VEGFA in PD-L1-silenced breast cancer cells and control cells. **d** HUVECs migration assay to investigate the effect of conditional medium from PD-L1-silenced breast cancer cells and control cells on the migration ability of HUVECs. Scale bars, 50 µm. **e** Statistical analysis of the HUVECs migration assay. *n* = 3. Data are presented as means ± SD. Two-tailed unpaired Student’s *t*-test. **f** HUVECs tube formation assay to investigate the effect of conditional medium from PD-L1 silenced breast cancer cells and control cells on HUVECs tubule formation. Scale bars, 50 µm. **g** Statistical analysis of the HUVECs tube formation assay. *n* = 3. Data are presented as means ± SD. Two-tailed unpaired Student’s *t*-test. **h** Schematic diagram of pro-angiogenic mechanism of nPD-L1 in UM. We demonstrated that PD-L1 nuclear translocation in UM facilitated angiogenesis by coactivating *EGR1* expression with p-STAT3. This elevated nPD-L1 level was mediated by HDAC2-dependent deacetylation in UM cells.

We then explored whether the promotion of EGR1-mediated angiogenesis by nPD-L1 is context-dependent (a special case in UM) or a wide-ranged function in cancers. Similar to what demonstrated by the previous nPD-L1 study^3^, we found that the mRNA levels of *EGR1* and *VEGFA* were decreased in PD-L1 knockout breast cancer cell (Fig. 8a, GSE134510). Concordantly, we also observed evident PD-L1 signals in the promoter region of *EGR1* in breast cancer cells (Fig. 8b, GSE146648)^3^. Moreover, we found that the PD-L1-deficient breast cancer cells (MDA-MB-231 and MDA-MB-436) exhibited decreased levels of EGR1 and VEGFA (Fig. 8c). Further functional experiments showed that PD-L1-deficient breast cancer cells presented a compromised capacity of promoting HUVEC migration (Fig. 8d, e) and tube formation (Fig. 8f, g). In addition, we reintroduced PD-L1^WT^ and PD-L1^K263R^ into PD-L1-silenced breast cancer cells (MDA-MB-231). Notably, increased EGR1 and VEGFA levels were observed after reintroducing PD-L1^K263R^, compared with the exogenous expression of PD-L1^WT^ (Supplementary Fig. S17a). In addition, HUVEC migration (Supplementary Fig. S17b, c) and tube formation (Supplementary Fig. S17d, e) assays showed that PD-L1^K263R^ exhibited increased capacity of promoting angiogenesis compared with PD-L1^WT^. Conclusively, these data suggested that the pro-vasculogenic effect of deacetylation-dependent nuclear transportation of PD-L1 might also be applied across multiple tumors, which warrants future validations.

## Discussion

The advent of immunotherapy against PD1/PD-L1 has dramatically improved the therapeutic algorithms for skin cutaneous melanoma and mucosal melanoma^37^. However, the specific mechanism underlying the poor response to anti-PD1/PD-L1 immunotherapy in UM, despite there are quite a number of PD-L1-positive cases, remains enigmatic. Herein, we demonstrated that UM presented elevated nPD-L1 levels, which was significantly different from the distribution patterns of PD-L1 in SKCM and mucosal melanoma. Notably, HDAC2-dependent deacetylation promoted the nuclear distribution of nPD-L1, which further facilitated angiogenesis by recruiting p-STAT3 to the target locus and activating EGR1 expression (Fig. 8h).

Of note, emerging studies have observed that PD-L1 translocates into the nucleus and plays immune-independent roles in tumorigenesis, such as promoting proliferation, metastasis, or immunotherapy resistance^3–5,38^. Intriguingly, our study revealed for the first time that nPD-L1 triggered angiogenesis through transcriptional activation of *EGR1* in UM, which expanded our understanding of the function of PD-L1. In addition, both published data^3^ and our experimental validation showed that this is also the case in breast cancer cells. Thus, the pro-angiogenic effect of PD-L1 might also be applicable to a variety of tumors. It was reported that nPD-L1 promotes a switch from apoptosis to pyroptosis under hypoxia conditions^5^. In this study, however, we didn’t observe any evidence for the change of pyroptosis/necrosis after silencing PD-L1, indicating that the biological and molecular functions of nPD-L1 may be different in different cancer cells.

Extensive angiogenesis supports nutrient supplementation in malignancies and serves as a hallmark in cancers^39,40^. Since the uveal membrane is a vascular layer formed by ECs, crosstalk between ECs and tumors is essential for the oncogenesis of UM^26,41^. Higher vascular density in UM tumors was reported to be associated with greater aggressiveness and worse prognosis^42,43^. Here, we proposed that nuclear PD-L1 was the trigger of angiogenesis in UM. Interestingly, enhanced angiogenesis could potentially promote the filtration of antitumor immune cells, which could be blocked by membrane PD-L1. Herein, we propose a multifaceted function of PD-L1 that serves as an endogenous accelerator of cancer angiogenesis, which provides a novel understanding of the crosstalk between angiogenesis and immune escape during carcinogenesis.

It should be noted that when combined with histone deacetylase inhibitors, anti-PD1/PD-L1 immunotherapy achieved durable responses in metastatic UM patients in a multicenter phase II study^44^. This study indicates that HDACi functions as a sensitizer for anti-PD1/PD-L1 immunotherapy. Our study added mechanical support that HDACi enhanced the efficacy of PD-1/PD-L1 blockade by elevating PD-L1 acetylation and reducing its nuclear proportion. Therefore, anti-PD1/PD-L1 therapy combined with HDACi leads to a more favorable outcome for UM patients.

In summary, our work unraveled the enigma of the disappointing efficacy of PD-1/PD-L1 blockade in UM. We also bridged angiogenesis and immunosuppression, which provided a novel understanding of PD-L1 regulation in tumorigenesis. Given that HDAC2-mediated PD-L1 deacetylation enhances its nuclear translocation, UM patients may benefit from PD-1/PD-L1 blockade and HDACi combination therapy.

Three limitations of our study should be addressed in the future: First, the association between nPD-L1 and driver mutation background remains unclear. Notably, several breast cancer cell, colon cancer cell and lung cancer cell lines presented robust nPD-L1 signals^3,38^, which were free of classic mutations (e.g., GNA11/GNAQ/BAP1) in UM. Further explorations are required in larger cohorts to reveal the mechanism underlying the crosstalk between certain genetic background and nPD-L1 signaling. Second, the comparison of nPD-L1 signal between the ‘responders’ and ‘non-responders’ towards anti-PD1/PD-L1 immunotherapy has not been revealed. This analysis would strengthen our assumption that elevated nPD-L1 contributes to unfavorable clinical efficacy of anti-PD1/PD-L1 immunotherapy in UM. Third, it’s important to apply syngeneic immunocompetent in vivo models to study both angiogenic and immunity-related effects of PD-L1/nPD-L1 in the future.

## Methods

### Patients and specimens

We collected 30 UM and 42 CoM tissues from the Department of Ophthalmology, Shanghai Ninth People’s Hospital, Shanghai Jiao Tong University School of Medicine, for IF staining. Clinical samples were used only with patient consent and the approval of the institutional research ethics committee. For SKCM, a commercial tissue chip was purchased from US Biomax (Supplementary Table S1).

### Ethics approval and consent to participate

Written informed consent for this study was obtained from all patients in Shanghai Ninth People’s Hospital, Shanghai Jiao Tong University School of Medicine. The process followed the tenets of the Declaration of Helsinki and was approved by the ethics committee of Shanghai Ninth People’s Hospital, Shanghai Jiao Tong University School of Medicine (approval No. SH9H-2019T185-2).

### Cell culture

The human UM cell lines MUM2B, OCM1, OMM1, OMM2.3, MEL290, and 92.1 were kindly provided by Professor John F. Marshall (Tumor Biology Laboratory, Cancer Research UK Clinical Center, John Vane Science Centre, London, UK) and Professor Martine J. Jager (Department of Ophthalmology, Leiden University Medical Center, Leiden, The Netherlands). The human CoM cell lines CRMM1, CRMM2, and CM2005.1 were also obtained from Professor Martine J. Jager (Department of Ophthalmology, Leiden University Medical Center, Leiden, The Netherlands). The human normal melanocyte cell line PIG1 was kindly supplied by the Department of Ophthalmology, Peking University Third Hospital. The HEK293T cell line was purchased from ATCC (Manassas, VA, USA). All cell lines were authenticated by short tandem repeat markers. For cell culturing, MUM2B, OCM1, and HEK293T cells were cultured in DMEM (Gibco) supplemented with 10% certified heat-inactivated fetal bovine serum (Gibco), penicillin (100 U/mL), and streptomycin (100 mg/mL) at 37 °C in a humidified 5% CO_2_ atmosphere. OMM1, OMM2.3, MEL290, and 92.1 cells were grown in RPMI 1640 medium (Gibco), and CRMM1, CRMM2, CM2005.1, and PIG1 cells were cultured in Ham’s F-12 K (Kaighn’s).

### IF assay

Deparaffinized and rehydrated tissue samples or cells seeded on glass slides were fixed with 4% formaldehyde for 30 min, blocked with 5% normal goat serum (Vector) for 1 h, and permeabilized with 0.5% Triton X-100 for 15 min. Thereafter, they were incubated with the primary antibodies at 4 °C overnight and the corresponding secondary antibodies for 1 h at room temperature. The nuclei were stained with DAPI (Sigma-Aldrich) for 30 min at room temperature. Finally, IF images were taken with a ZEISS Axio Scope A1 upright microscope. As described in Fig. 1a, the PD-L1-positive group was defined as more than 5% of the cells having PD-L1 signals. Then, in the PD-L1-positive group, we defined a PD-L1 ratio > 20% in the nucleus as the nuclear PD-L1-positive group, which was measured by ImageJ 1.52 software.

### Western blot

Briefly, cells were lysed with RIPA lysis buffer supplemented with cocktail (NCM Biotech, Suzhou, China) on a shaking bed at 4 °C for 20 min and centrifuged at 13,000× *g* at 4 °C for 30 min. Then, the protein supernatants were boiled with SDS loading buffer, separated by 7.5% (wt/vol) SDS-PAGE, and transferred to PVDF membranes (Millipore, USA). The membranes were blocked with 5% milk for 1 h and incubated with the primary antibodies at 4 °C overnight. Finally, the band signals were visualized by an Odyssey Infrared Imagining System (LI-COR, USA) after incubation with the appropriate fluorescent secondary antibodies (CST). The following primary antibodies were used in this study: anti-PD-L1 (CST, 13684 S), anti-EGR1 (CST, 4153 S), anti-VEGFA (Abcam, ab46154), anti-HDAC2 (CST, 5113 S), anti-STAT3 (Abcam, ab226942), anti-phospho-STAT3 (Tyr705) (CST, 9145 S), anti-β-actin (ABclonal, AC026), and anti-H3 (Proteintech, 17168-1-AP).

### Nuclear/cytosolic fractionation

The cellular nuclear and cytosolic fractions were isolated using a nuclear and cytoplasmic protein extraction kit (Beyotime, P0028) according to the manufacturer’s instructions. Briefly, 20 µL of cell precipitate was suspended in 200 µL of cytoplasmic protein extraction reagent A and incubated for 15 min on ice followed by adding 10 µL of cytoplasmic protein extraction reagent B. After incubating for 1 min, the cells were centrifuged for 5 min at 12,000× *g*. The supernatant was harvested for the cytoplasmic component, and the pellet was resuspended in 50 µL of nuclear protein extraction reagent for the nuclear fraction. The isolated fractions were processed for western blot analysis.

### qRT-PCR

Total RNA was isolated from cultured cell samples by the EZpress RNA Purification Kit (EZBioscience, B0004) according to the manufacturer’s instructions. Then, cDNA was prepared by the PrimeScript RT reagent kit (TaKaRa Bio, RR036A), followed by qRT-PCR using SYBR Green PCR master mix (Life Technologies, A25742). We used ACTB as an endogenous reference for PCR product quantification and normalization. The primers for the qRT-PCR analysis are listed in Supplementary Table S2.

### HUVEC migration assay

HUVECs suspended in serum-free medium were placed in the upper chamber of a 24-well Transwell system (Corning) with polycarbonate filters (8-μm pores, Corning). Then, 500 μL of conditional medium was added to the lower chamber. After 12 h of incubation, the cells that migrated to the bottom of the membranes were stained with 0.25% crystal violet for 20 min, followed by imaging and counting.

### HUVEC tube formation assay

48-well plates were precoated with 150 μL precooled Matrigel (Corning, 354234) per well and polymerized at 37 °C for 30 min. HUVECs (1 × 10^4^ cells) suspended in 200 μL of conditional medium were seeded into each well and further cultured for 3 h. Then, the fields of tube structure were randomly chosen and photographed for quantification.

### CAM assay

The CAM assay was performed using fertilized eggs on the eighth day. A round window was opened on the air sac of the eggshell, and the surface of the dermic sheet was removed to expose the CAM. Immediately, a plastic ring was placed on the CAM, and 150 μL of conditional medium was carefully dropped onto the plastic ring. Then, the window was sealed with tape. After 3 days of incubation, images of CAMs were taken to evaluate the number of blood vessels around the paper disc.

### Animal experiments

Four-week-old male BALB/c nude mice were used for UM orthotopic xenograft experiments in a specific pathogen-free animal room. Nude mice were pre-perforated in sclera by a sharp 30-gauge injection needle after anesthetization. UM cells were then injected into the choroid through the hole by a 33-gauge blunt-end microinjection needle (Hamilton, 7803-05). Ophthalmic bacitracin ointment was applied to the injected eyes. All mice were finally sacrificed by cervical dislocation after 3 weeks for tumor formation analysis. The animal experiments were all approved by the Animal Care and Use Committee of Shanghai Ninth People’s Hospital, Shanghai Jiao Tong University School of Medicine (ethical committee approval No. SH9H-2020-A686-1 and SYXK (Shanghai) 2016-0016).

### High throughput CUT&Tag

The CUT&Tag assay was carried out as previously described^45^ with some modifications by Jiayin Biotechnology Ltd. (Shanghai, China). In brief, nuclei purified from tumor cells were washed and incubated with concanavalin A-coated magnetic beads. Then, the nuclei-bound beads were washed and incubated with the primary antibody or control IgG overnight at 4 °C on a rotating platform. After incubation with the secondary antibody, the beads were treated with the proteinA-Tn5 transposome for 1 h at room temperature. Next, they were resuspended in tagmentation buffer, and DNA fragments were purified by phenol-chloroform-isoamyl alcohol extraction and ethanol precipitation. Finally, the amplified libraries were sequenced on an Illumina NovaSeq 6000 using 150 bp paired-end sequencing according to the manufacturer’s instructions.

### RNA-seq

To perform RNA-seq, total RNA was isolated from the cultured cells by TRIzol reagent (Invitrogen, Carlsbad, CA, USA). The integrity of the RNA was confirmed by a 2100 Bioanalyzer (Agilent Technologies, USA), and the concentration was measured by a Qubit 2.0 fluorometer with a Qubit RNA assay kit (Life Technologies, Carlsbad, CA, USA). Then, sequencing libraries were generated using an Illumina TruSeq RNA Sample Prep Kit (San Diego, CA, USA). The libraries were finally sequenced on the Illumina HiSeq 2500 platform.

### ChIP-qPCR

ChIP assays were performed using a ChIP Kit (Millipore, 17-371) according to the manufacturer’s instructions. Briefly, the cells were fixed, lysed and sonicated for 8 min (10 s on and 15 s off) on ice. To precipitate the DNA fragments, sonicated chromatin fragments were diluted and incubated with Protein G agarose beads and the primary antibody or control IgG overnight at 4 °C on a rotating platform. Then, the DNA–protein-bead complexes were decrosslinked and treated with proteinase K. Finally, the DNA fragments were purified for qPCR analysis. The primers for the ChIP-qPCR analysis are listed in Supplementary Table S2.

### TCGA dataset

We queried TCGA (www.cbioportal.org) and GEPIA (gepia.cancerpku.cn), which provide tumor transcriptional data and follow-up information, to validate the prognostic significance of PD-L1 and its expression correlation with EGR1 and VEGFA in UM and SKCM.

### Statistical analysis

Statistical analysis was performed using GraphPad Prism 8 software. Quantification data are presented as means ± SD of biological triplicates, and the differences between two groups were compared using unpaired two-tailed Student’s *t*-test. Survival curves were calculated by the Kaplan–Meier method. *P* < 0.05 was considered statistically significant and asterisks denoted statistical significance (*P < 0.05, **P < 0.01, ***P < 0.001).

## Supplementary information

Supplementary information

## Data Availability

Raw data including RNA-seq and CUT&Tag data, have been deposited in the Gene Expression Omnibus database under accession numbers GSE202884, GSE202394 and GSE202883.
