# Supplementary material for: Nuclear PD-L1 promotes EGR1-mediated angiogenesis and accelerates tumorigenesis
Source: Cell Discov. 2023 Mar 28;9:33. doi: 10.1038/s41421-023-00521-7 (PMC10050073; doi:10.1038/s41421-023-00521-7)
Supplement: Supplementary file 1 — Supplementary information [file 41421_2023_521_MOESM1_ESM.pdf]

Supplementary information

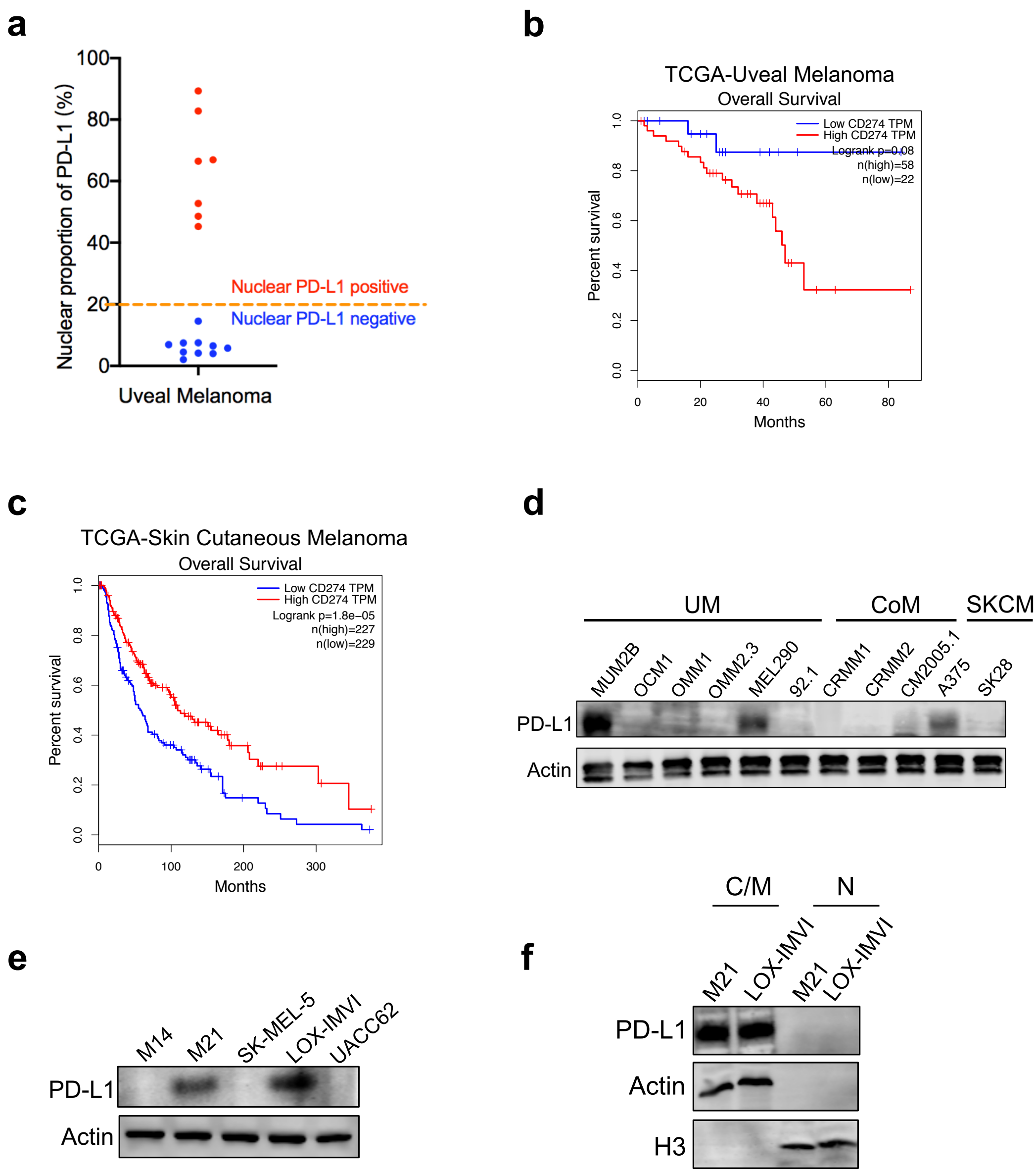

Supplementary Fig. 1. PD-L1 expression in uveal melanoma. a Definition of nPD-L1 positive and negative groups according to the 20% proportion of nPD-L1. **b**, **c** Kaplan-Meier analysis of the overall survival of UM (**b**) and SKCM (**c**) patients with low and high PD-L1 expression levels queried by TCGA database. **d** Western blot analysis of PD-L1 expression levels in UM (MUM2B, OCM1, OMM1, OMM2.3, MEL290 and 92.1), CoM (CRMM1, CRMM2 and CM2005.1), and SKCM (A375 and SK28) cell lines. **e** Western Blot detection of PD-L1 in SKCM (M14, M21, SK-MEL-5, LOX-IMVI and UACC62) cells. **f** Western blot analysis of cytoplasmic/membrane (C/M) and nuclear (N) fractions of PD-L1 in above SKCM cells.

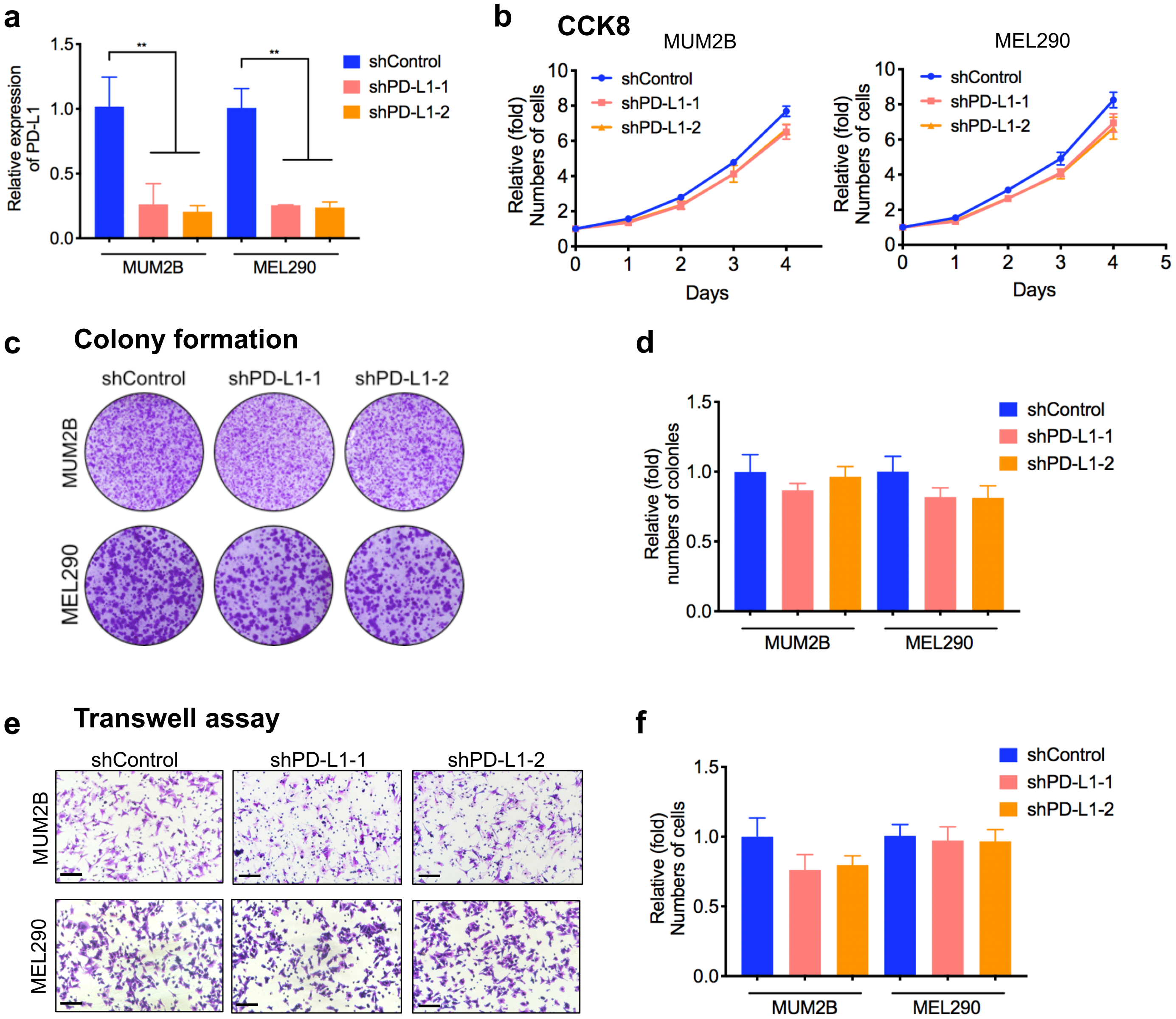

Supplementary Fig. 2. PD-L1 knockdown had no significant effect on uveal melanoma cell proliferation and migration. a Stable silencing of PD-L1 in MUM2B and MEL290 cells verified by RT-PCR. **b** CCK8 assay that investigates the effect of PD-L1 silencing on cell proliferation ability. **c** Colony formation assay that investigates the effect of PD-L1 silencing on cell colony formation ability. **d** Statistical analysis of the colony formation assay. $n = 3$. Data are presented as means ± SD. Two-tailed unpaired Student's *t*-tests. **e** Tranwell assay that investigates the effect of PD-L1 silencing on cell migration ability. **f** Statistical analysis of the tranwell assay. $n = 3$. Data are presented as means ± SD. Two-tailed unpaired Student's *t*-tests.

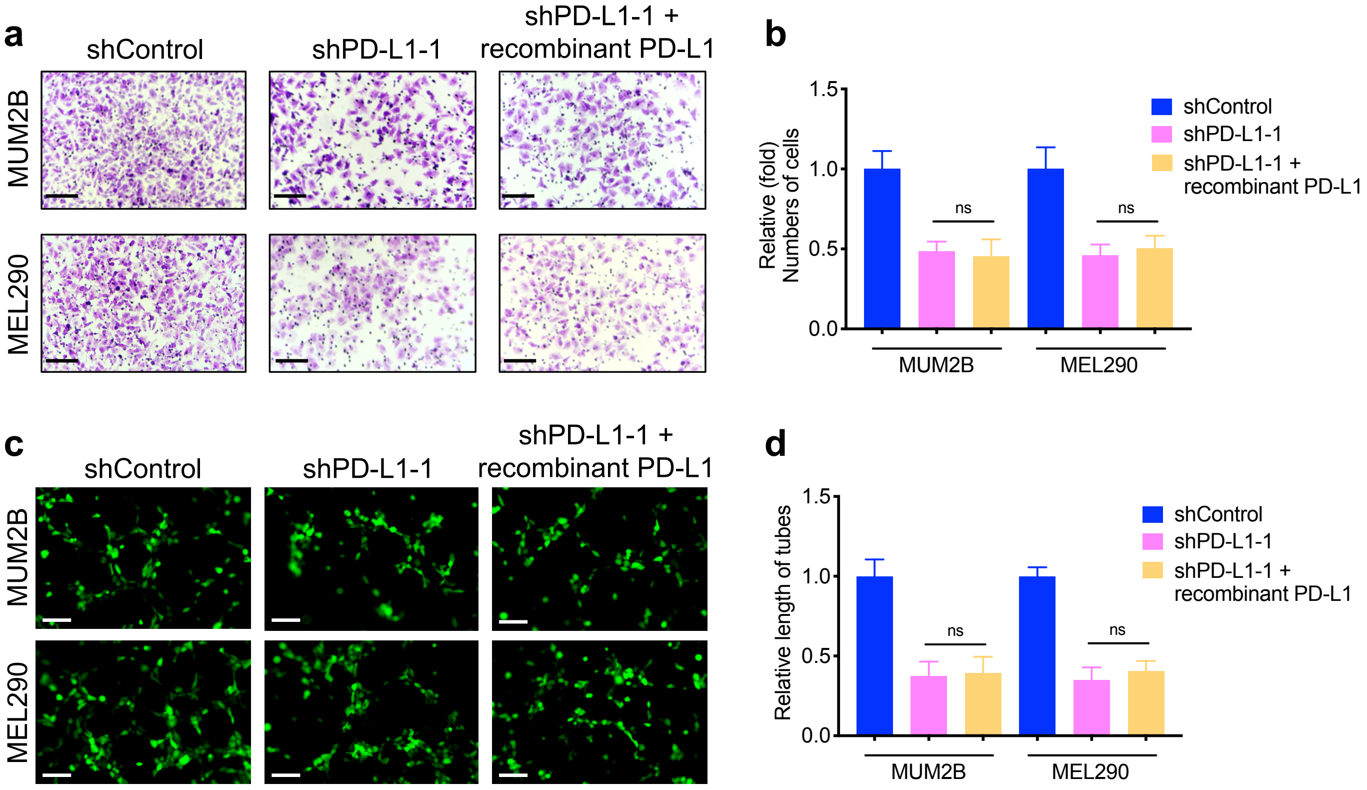

Supplementary Fig. 3. Recombinant PD-L1 failed to restore angiogenesis in PD-L1-depleted cells. a HUVEC migration assay that investigates the effect of recombinant PD-L1 in PD-L1-depleted cells on the migration ability of HUVECs. Scale bars, 50 μm. **b** Statistical analysis of the HUVEC migration assay. $n = 3$. Data are presented as means ± SD. Two-tailed unpaired Student's *t*-tests. **c** HUVEC tube formation assay that investigates the effect of recombinant PD-L1 in PD-L1-depleted cells on HUVEC tubule formation. Scale bars, 25 μm. **d** Statistical analysis of the HUVEC tube formation assay. $n = 3$. Data are presented as means ± SD. Two-tailed unpaired Student's *t*-tests.

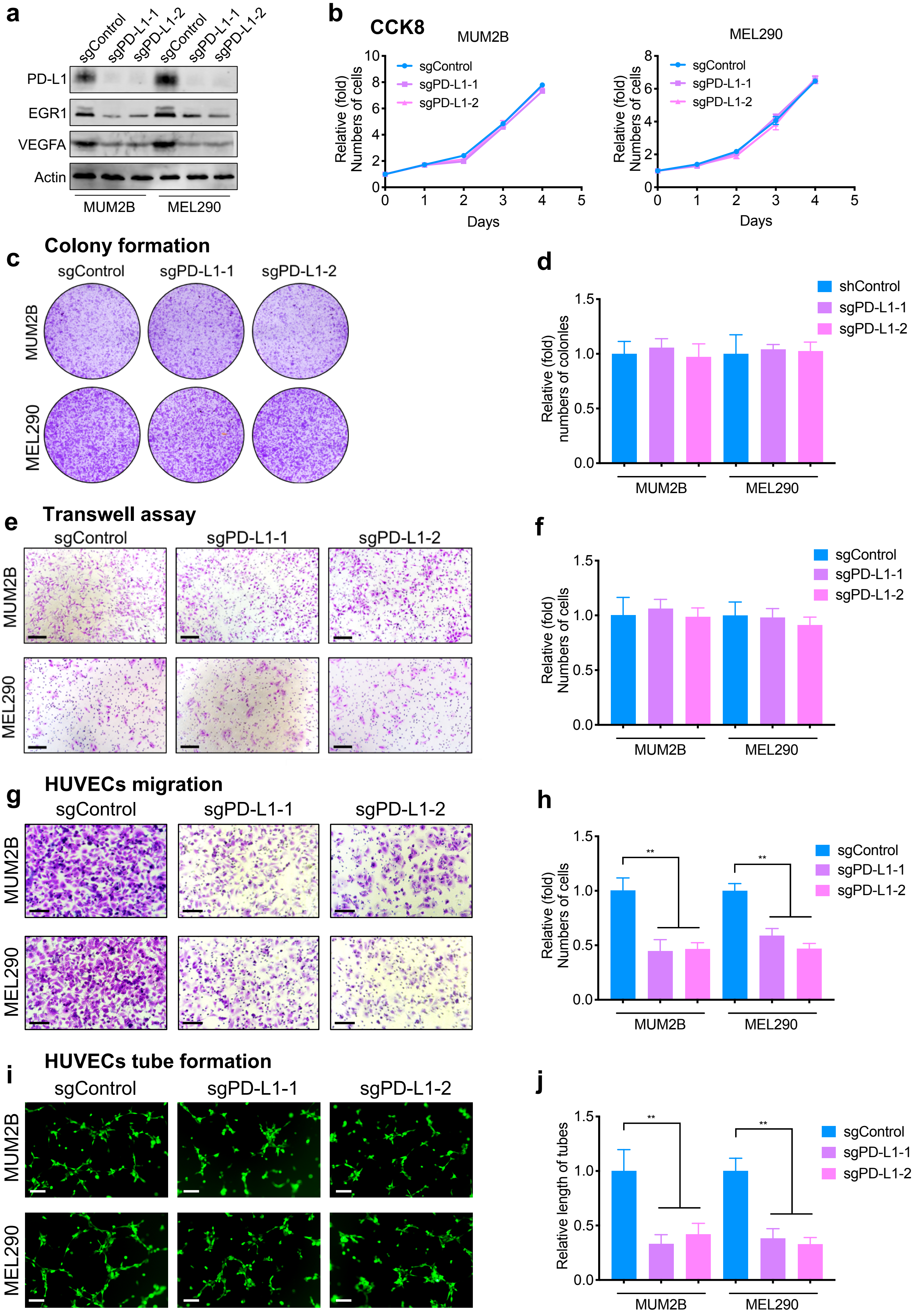
a
sgControl
sgPD-L1-1
sgPD-L1-2
sgControl
sgPD-L1-1
sgPD-L1-2
PD-L1
EGR1
VEGFA
Actin
MUM2B
MEL290
b
CCK8
MUM2B
MEL290
Relative (fold) Numbers of cells
sgControl
sgPD-L1-1
sgPD-L1-2
Days
c
Colony formation
sgControl
sgPD-L1-1
sgPD-L1-2
MUM2B
MEL290
d
Relative (fold) numbers of colonies
shControl
sgPD-L1-1
sgPD-L1-2
MUM2B
MEL290
e
Transwell assay
sgControl
sgPD-L1-1
sgPD-L1-2
MUM2B
MEL290
f
Relative (fold) Numbers of cells
sgControl
sgPD-L1-1
sgPD-L1-2
MUM2B
MEL290
g
HUVECs migration
sgControl
sgPD-L1-1
sgPD-L1-2
MUM2B
MEL290
h
Relative (fold) Numbers of cells
sgControl
sgPD-L1-1
sgPD-L1-2
**
**
MUM2B
MEL290
i
HUVECs tube formation
sgControl
sgPD-L1-1
sgPD-L1-2
MUM2B
MEL290
j
Relative length of tubes
sgControl
sgPD-L1-1
sgPD-L1-2
**
**
MUM2B
MEL290

Supplementary Fig. 4. PD-L1 knockout did not significantly alter proliferation and migration in uveal melanoma cells. **a** Stable knockout of PD-L1 in MUM2B and MEL290 cells verified by Western blot. **b** CCK8 assay that investigates the effect of PD-L1 knockout on cell proliferation ability. **c** Colony formation assay that investigates the effect of PD-L1 knockout on cell colony formation ability. **d** Statistical analysis of the colony formation assay. $n = 3$. Data are presented as means ± SD. Two-tailed unpaired Student's *t*-tests. **e** Tranwell assay that investigates the effect of PD-L1 knockout on cell migration ability. Scale bars, 50 μm. **f** Statistical analysis of the tranwell assay. $n = 3$. Data are presented as means ± SD. Two-tailed unpaired Student's *t*-tests. **g** HUVEC migration assay that investigates the effect of conditional medium from PD-L1-knockout cells and control cells on the migration ability of HUVECs. Scale bars, 50 μm. **h** Statistical analysis of the HUVEC migration assay. $n = 3$. Data are presented as means ± SD. Two-tailed unpaired Student's *t*-tests. **i** HUVEC tube formation assay that investigates the effect of conditional medium from PD-L1-knockout cells and control cells on HUVEC tubule formation. Scale bars, 50 μm. **j** Statistical analysis of the HUVEC tube formation assay. $n = 3$. Data are presented as means ± SD. Two-tailed unpaired Student's *t*-tests.

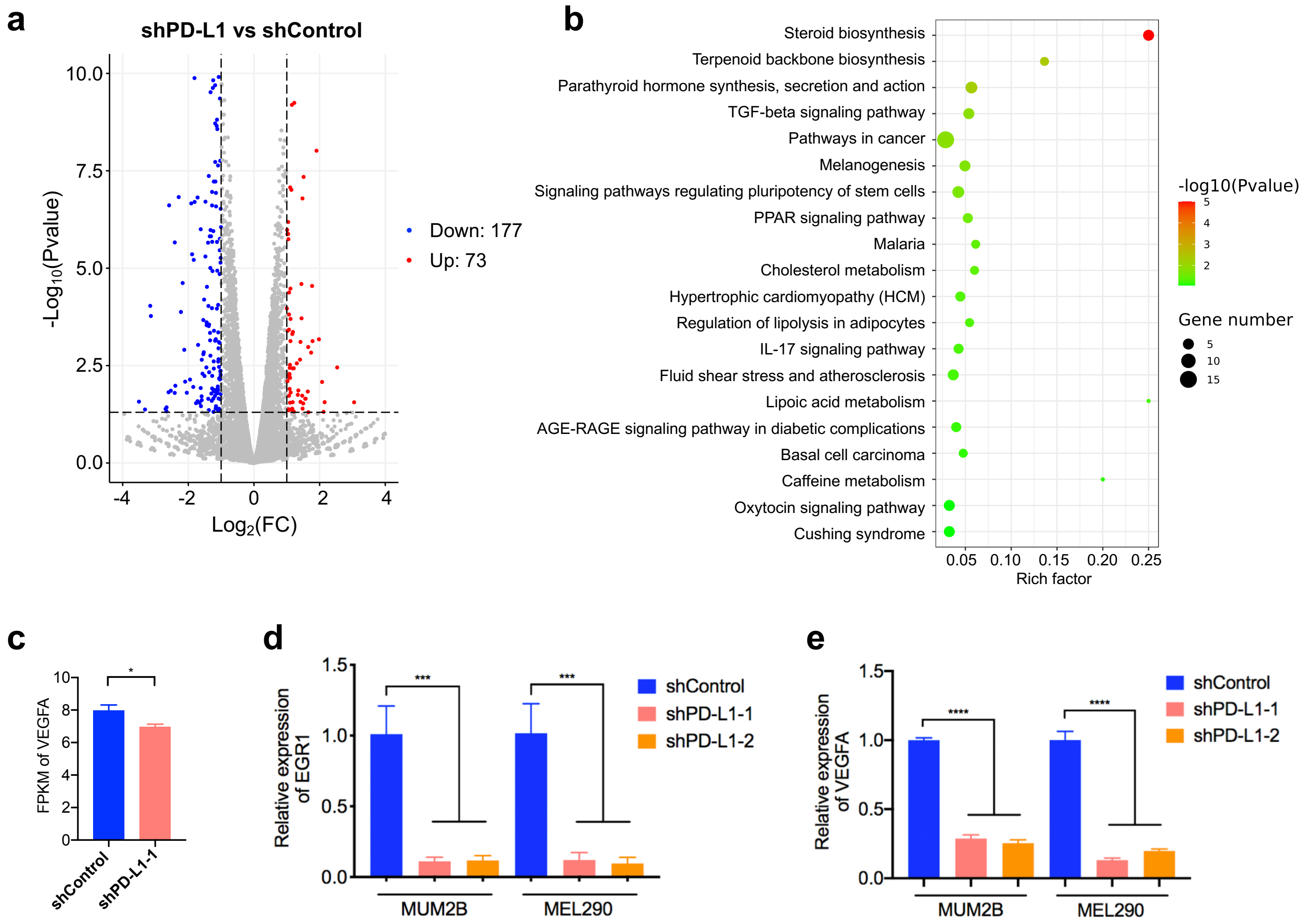

Supplementary Fig. 5. RNA-seq of PD-L1 silencing in uveal melanoma. a Volcano plot of dysregulated genes in PD-L1 silenced cells. **b** Kyoto Encyclopedia of Genes and Genomes (KEGG) analysis of dysregulated genes in PD-L1 silenced cells. **c** FPKM for VEGFA expression from RNA-seq analysis of PD-L1 silenced cells and control cells. **d**, **e** RT-PCR detecting relative EGR1 (**d**) and VEGFA (**e**) mRNA expression in MUM2B and MEL290 cells after PD-L1 knocking down. n = 3. Data are presented as means ± SD. Two-tailed unpaired Student's t-tests.

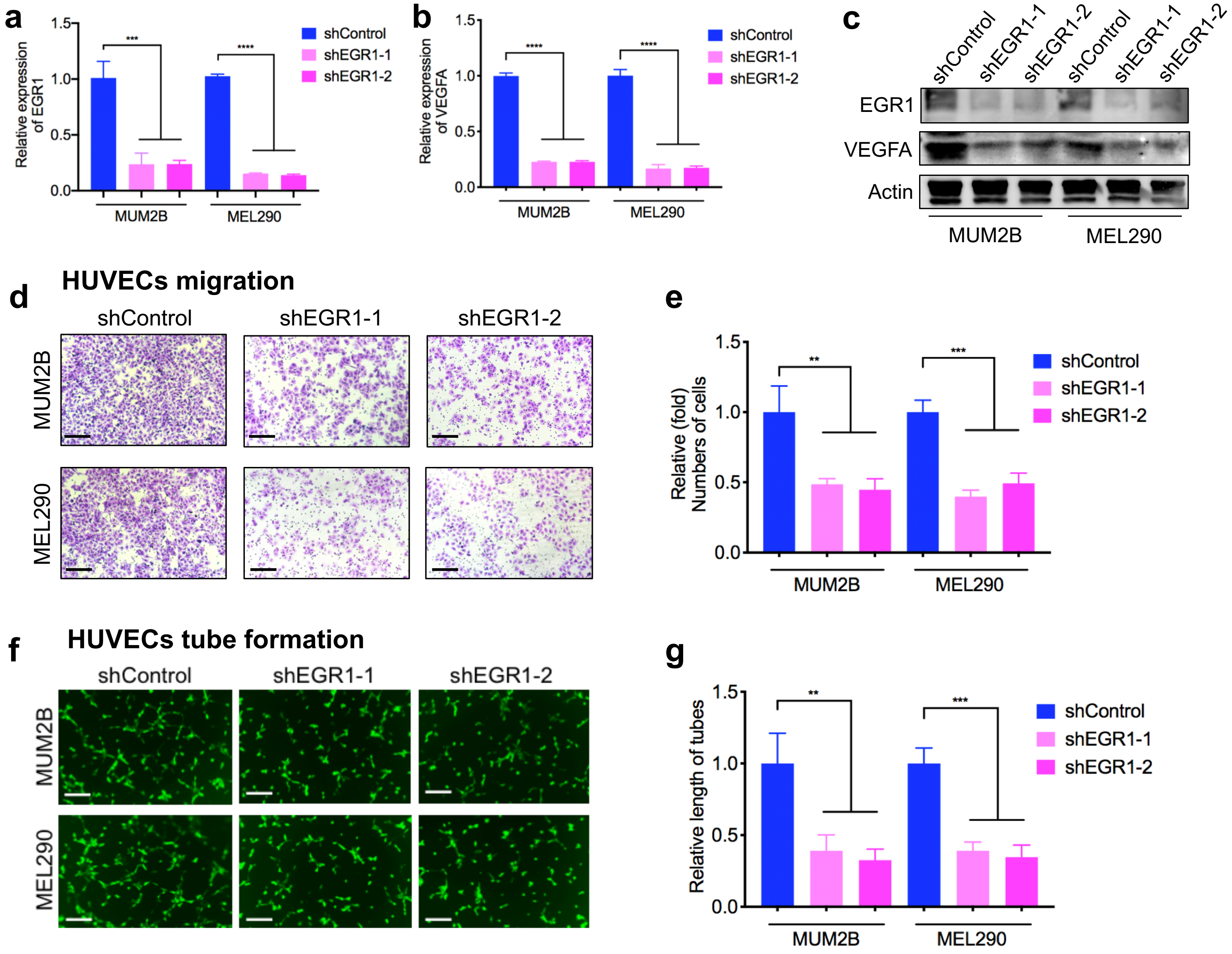

Supplementary Fig. 6. EGR1 facilitates angiogenesis in uveal melanoma. a, **b** RT-PCR detecting relative EGR1 (**a**) and VEGFA (**b**) mRNA expression in MUM2B and MEL290 cells after EGR1 knocking down. **c** Western blot images showing the protein levels of EGR1 and VEGFA in EGR1 silenced cells and control cells. **d** HUVECs migration assay that investigates the effect of conditional medium from EGR1 silenced cells and control cells on the migration ability of HUVECs. Scale bars, 50 μm. **e** Statistical analysis of the HUVECs migration assay. n = 3. Data are presented as means ± SD. Two-tailed unpaired Student's t-tests. **f** HUVECs tube formation assay that investigates the effect of conditional medium from EGR1 silenced cells and control cells on HUVECs tubule formation. Scale bars, 50 μm. **g** Statistical analysis of the HUVECs tube formation assay. n = 3. Data are presented as means ± SD. Two-tailed unpaired Student's t-tests.

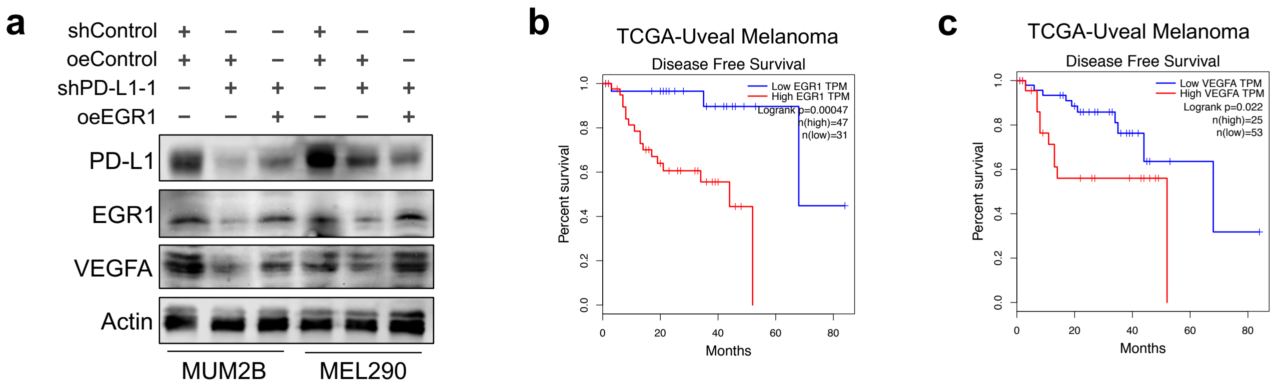

Supplementary Fig. 7. Validation of EGR1 restoration efficiency. a Western blot images showing the protein levels of PD-L1, EGR1 and VEGFA in PD-L1 silenced cells after EGR1 restoration. **b**, **c** Kaplan-Meier analysis of the disease free survival of UM patients with low and high EGR1 (**b**) and VEGFA (**c**) expression levels queried by TCGA database.

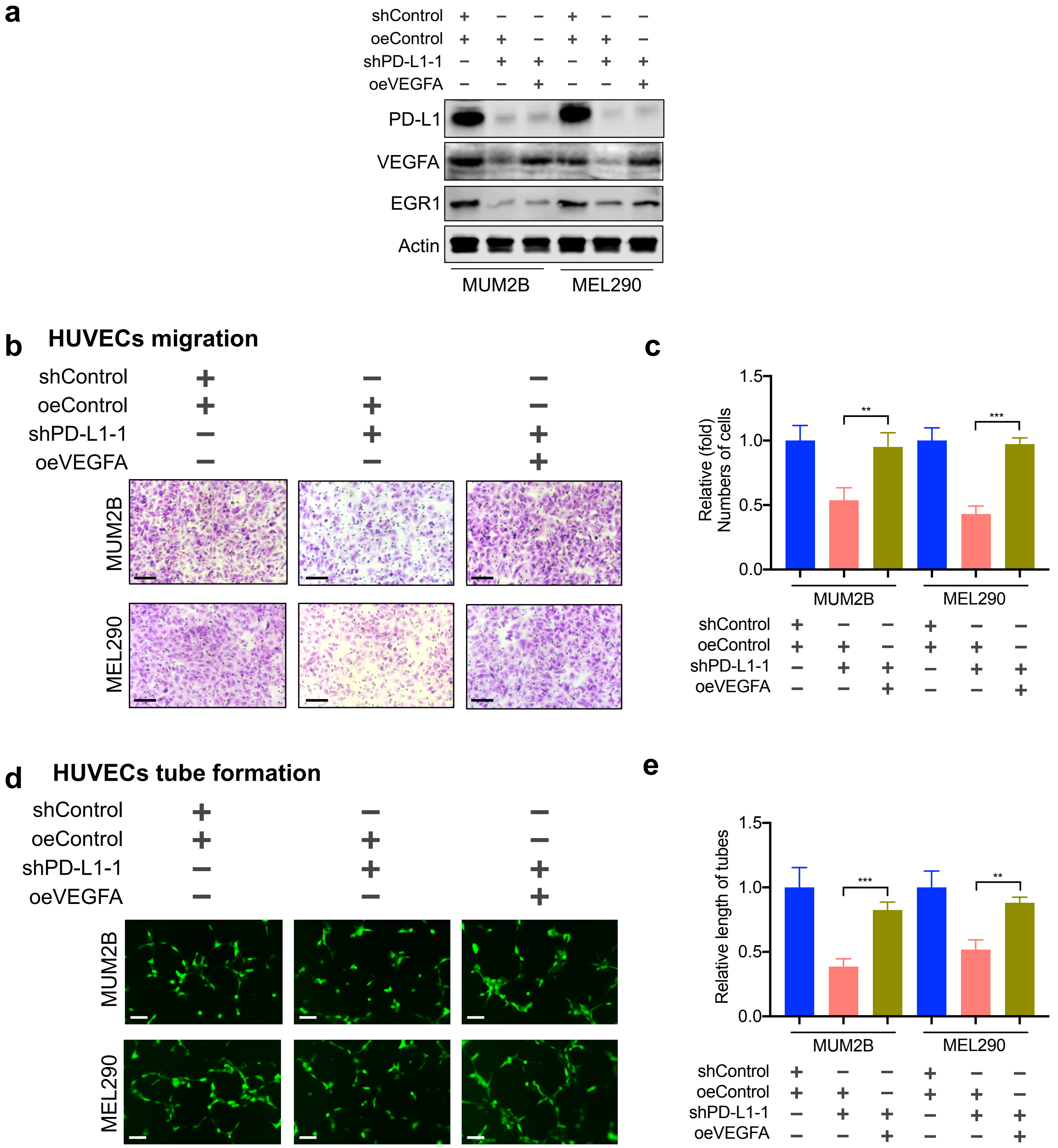

Supplementary Fig. 8. Restoration of VEGFA rescued pro-angiogenesis capacity in PD-L1-silenced cells. a Western blot images showing the protein levels of PD-L1, EGR1 and VEGFA in PD-L1 silenced cells after VEGFA restoration. **b** HUVEC migration assay that investigates the effect of VEGFA re-expression in PD-L1-silenced cells on the migration ability of HUVECs. Scale bars, 50 μm. **c** Statistical analysis of the HUVEC migration assay. $n = 3$. Data are presented as means ± SD. Two-tailed unpaired Student's *t*-tests. **d** HUVEC tube formation assay that investigates the effect of VEGFA re-expression in PD-L1-silenced cells on HUVEC tubule formation. Scale bars, 25 μm. **e** Statistical analysis of the HUVEC tube formation assay. $n = 3$. Data are presented as means ± SD. Two-tailed unpaired Student's *t*-tests.

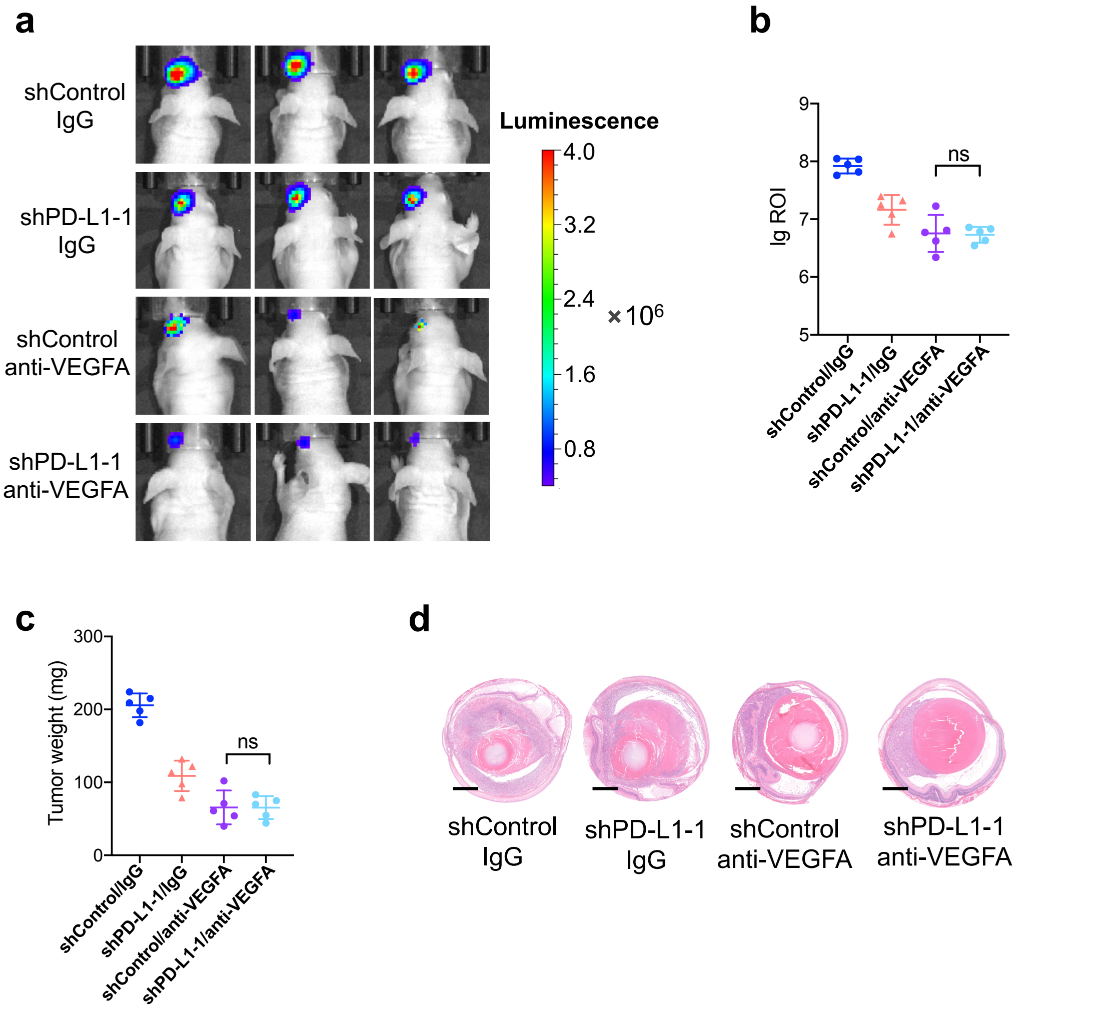

Supplementary Fig. 9. Combined anti-VEGF and PD-L1 silencing treatment in UM. a Representative bioluminescent images of UM orthotopic xenografts generated by tumor cells with indicated constructs and treated with anti-VEGFA. **b** Statistical analysis of the bioluminescent signals in UM orthotopic xenografts. n = 5. Data are presented as the means ± SD. Two-tailed unpaired Student's t-tests. **c** Statistical analysis of the tumor weight. n = 5. Data are presented as the means ± SD. Two-tailed unpaired Student's t-tests. **d** Representative images of H&E staining of UM orthotopic xenografts generated by tumor cells with indicated constructs and treated with anti-VEGFA.

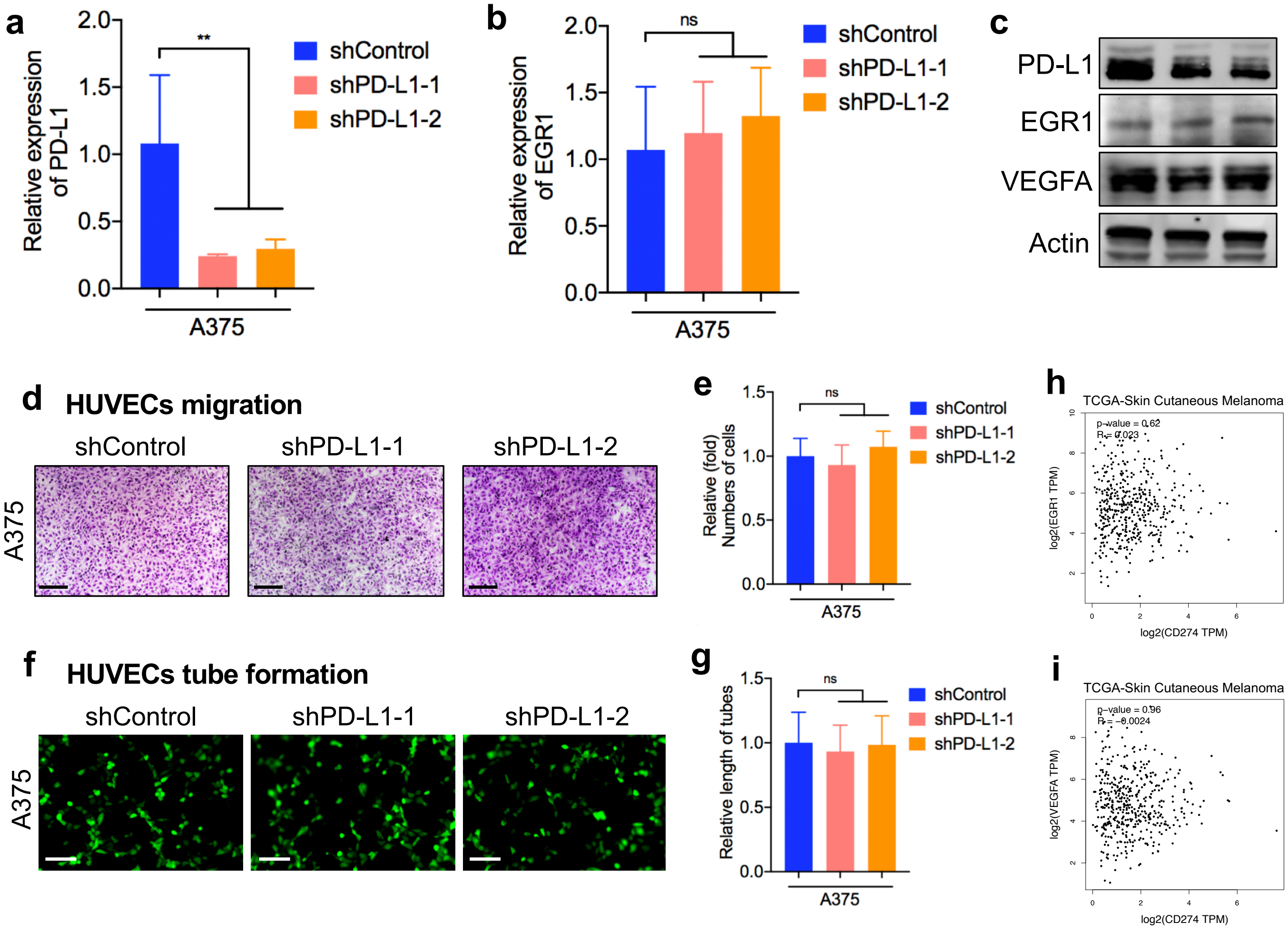

Supplementary Fig. 10. PD-L1 failed to facilitate angiogenesis in cutaneous melanoma. a, **b** RT-PCR detecting relative PD-L1 (**a**) and EGR1 (**b**) mRNA expression in A375 cells after PD-L1 knocking down. $n = 3$. Data are presented as means ± SD. Two-tailed unpaired Student's *t*-tests. **c** Western blot images showing the protein levels of PD-L1, EGR1 and VEGFA in A375 cells after PD-L1 knocking down. **d** HUVECs migration assay that investigates the effect of conditional medium from A375 cells after PD-L1 knocking down on the migration ability of HUVECs. Scale bars, 100 μm. **e** Statistical analysis of the HUVECs migration assay. $n = 3$. Data are presented as means ± SD. Two-tailed unpaired Student's *t*-tests. **f** HUVECs tube formation assay that investigates the effect of conditional medium from A375 cells after PD-L1 knocking down on HUVECs tubule formation. Scale bars, 25 μm. **g** Statistical analysis of the HUVECs tube formation assay. $n = 3$. Data are presented as means ± SD. Two-tailed unpaired Student's *t*-tests. **h**, **i** Gene expression correlation analysis of PD-L1 and EGR1 (**h**), PD-L1 and EGR1 (**i**) in SKCM patients queried by TCGA database.

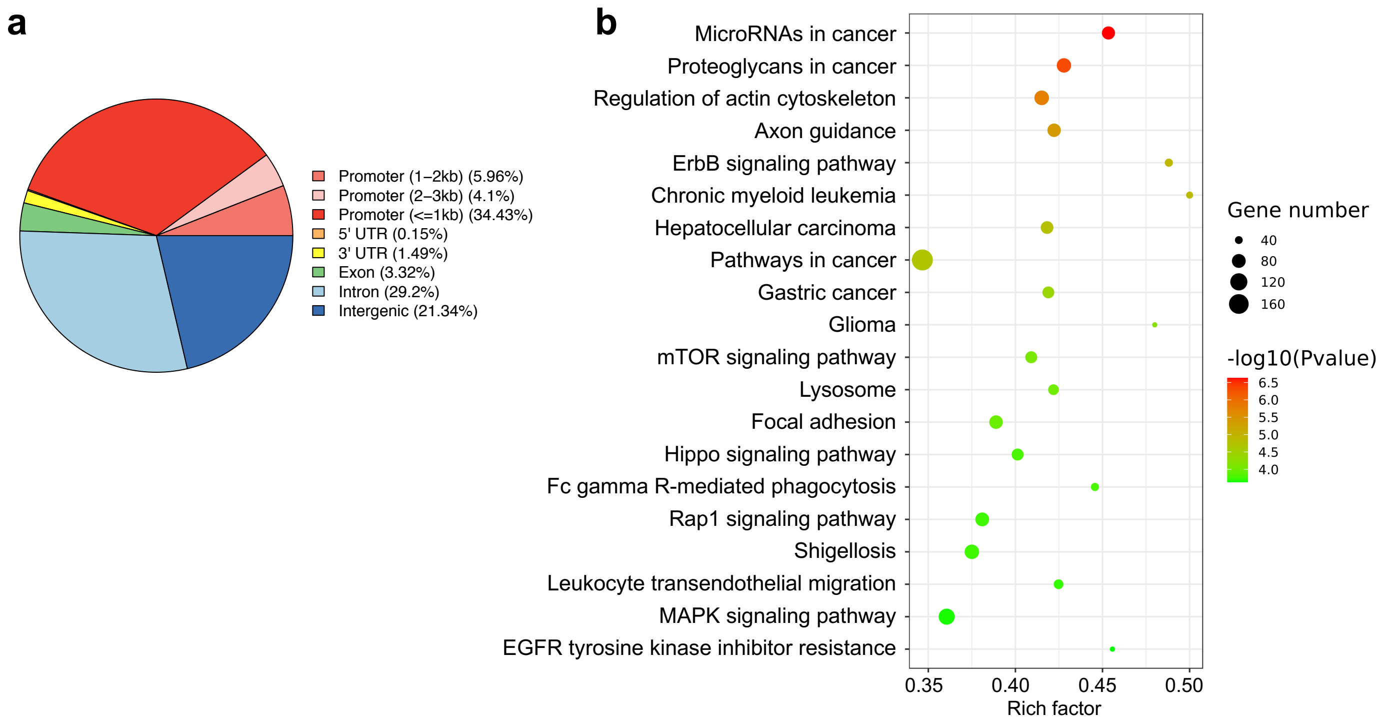

Supplementary Fig. 11. p-STAT3 CUT&Tag analysis. a The genomic distribution of down-regulated p-STAT3 CUT&Tag peaks in PD-L1 silenced cells. **b** Kyoto Encyclopedia of Genes and Genomes (KEGG) analysis shows that these down-regulated binding loci of p-STAT3 in PD-L1 silenced cells are associated with multiple oncogenic pathways.

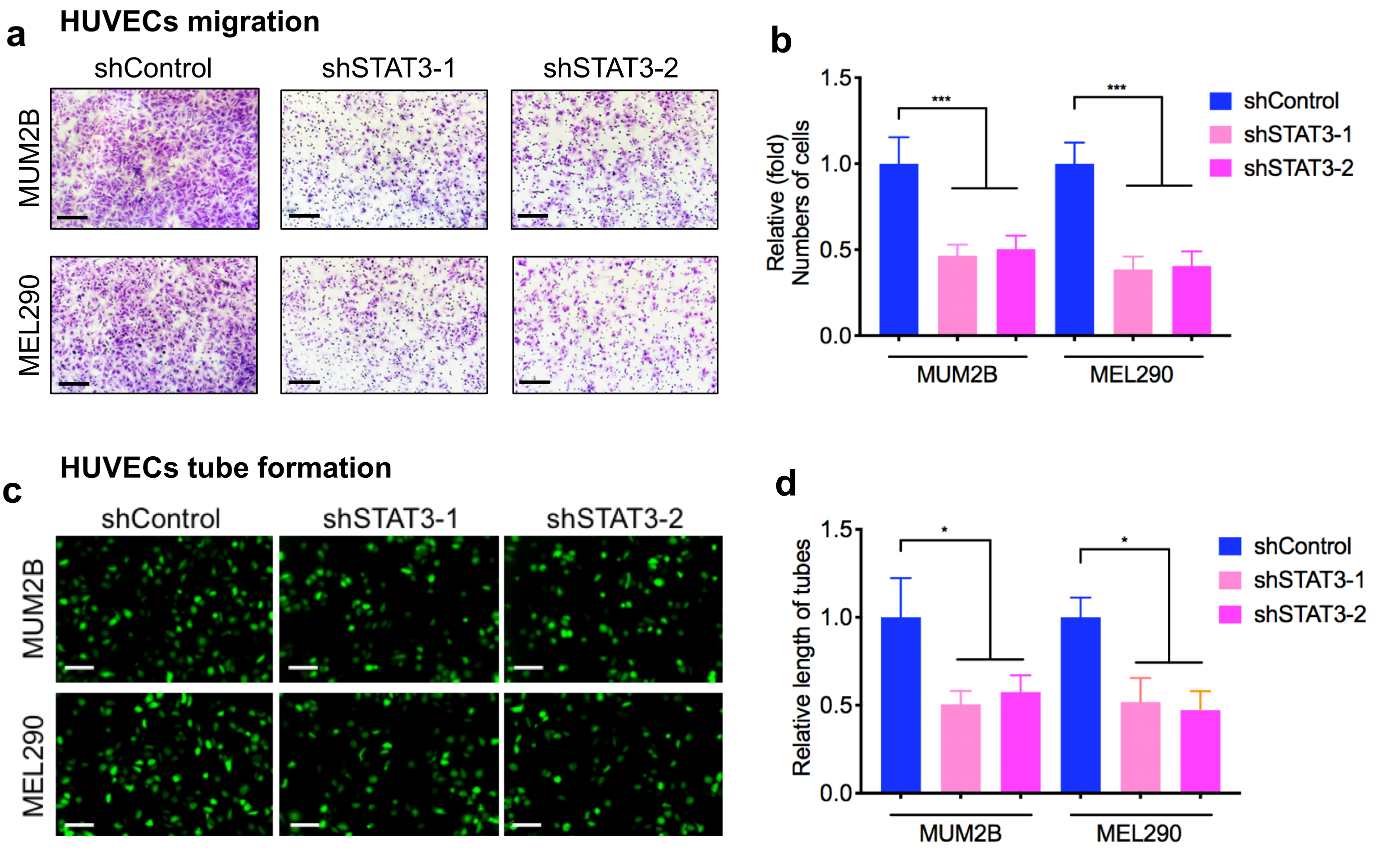

Supplementary Fig. 12. STAT3 knockdown inhibits angiogenesis in uveal melanoma. a HUVECs migration assay that investigates the effect of conditional medium from STAT3 silenced cells and control cells on the migration ability of HUVECs. Scale bars, 50 μm. **b** Statistical analysis of the HUVECs migration assay. $n = 3$. Data are presented as means ± SD. Two-tailed unpaired Student's *t*-tests. **c** HUVECs tube formation assay that investigates the effect of conditional medium from STAT3 silenced cells and control cells on HUVECs tubule formation. Scale bars, 25 μm. **d** Statistical analysis of the HUVECs tube formation assay. $n = 3$. Data are presented as means ± SD. Two-tailed unpaired Student's *t*-tests.

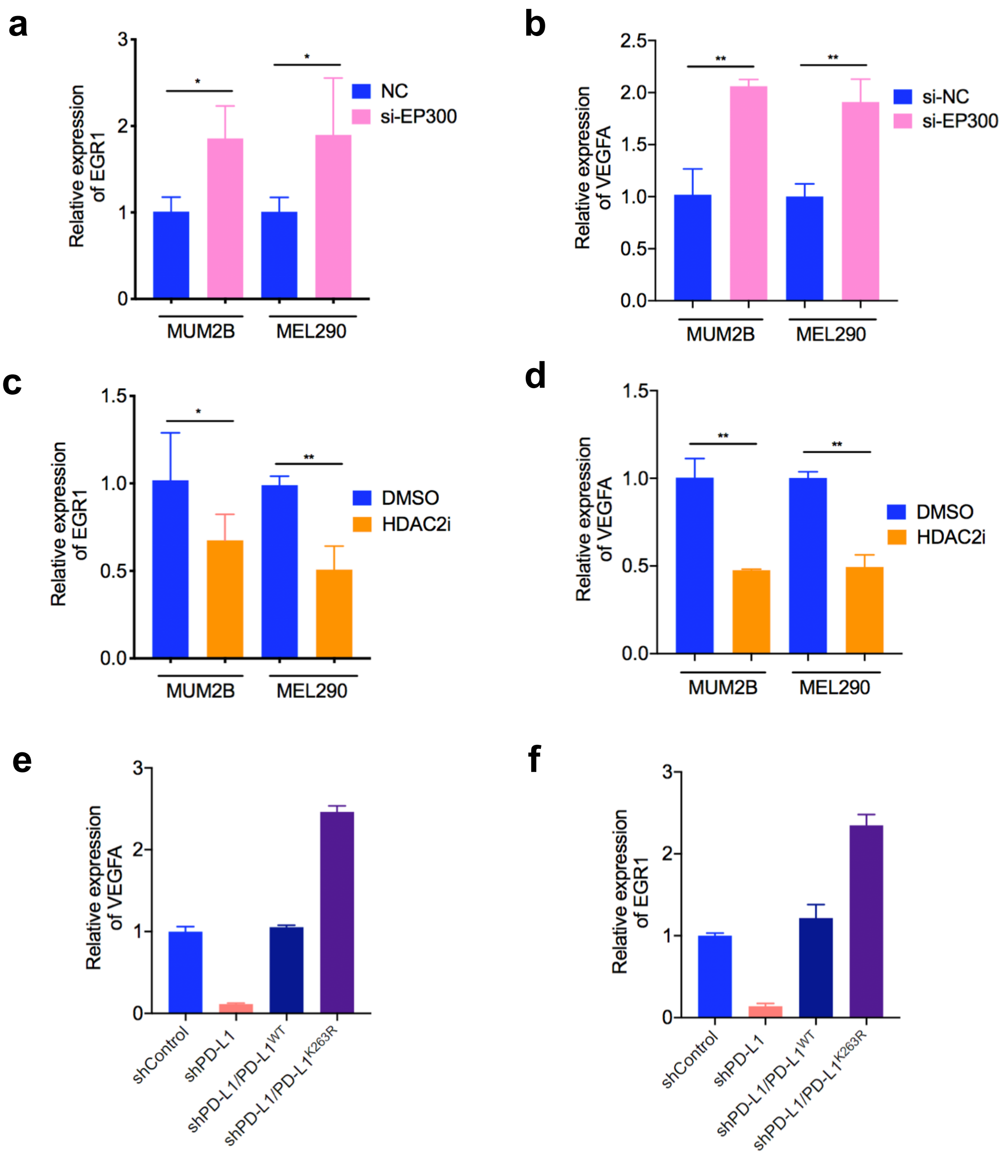

Supplementary Fig. 13. PD-L1^{K263} deacetylation promotes EGR1 and VEGFA expression in UM. a, **b** RT-PCR detecting relative EGR1 (**a**) and VEGFA (**b**) mRNA expression in MUM2B and MEL290 cells transduced with si-EP300. **c**, **d** RT-PCR detecting relative EGR1 (**c**) and VEGFA (**d**) mRNA expression in MUM2B and MEL290 cells treated with 50 μM HDAC2i (santacruzamate A) for 24 h. **e**, **f** RT-PCR detecting relative VEGFA (**e**) and EGR1 (**f**) mRNA expression in MUM2B cells treated with the indicated constructs.

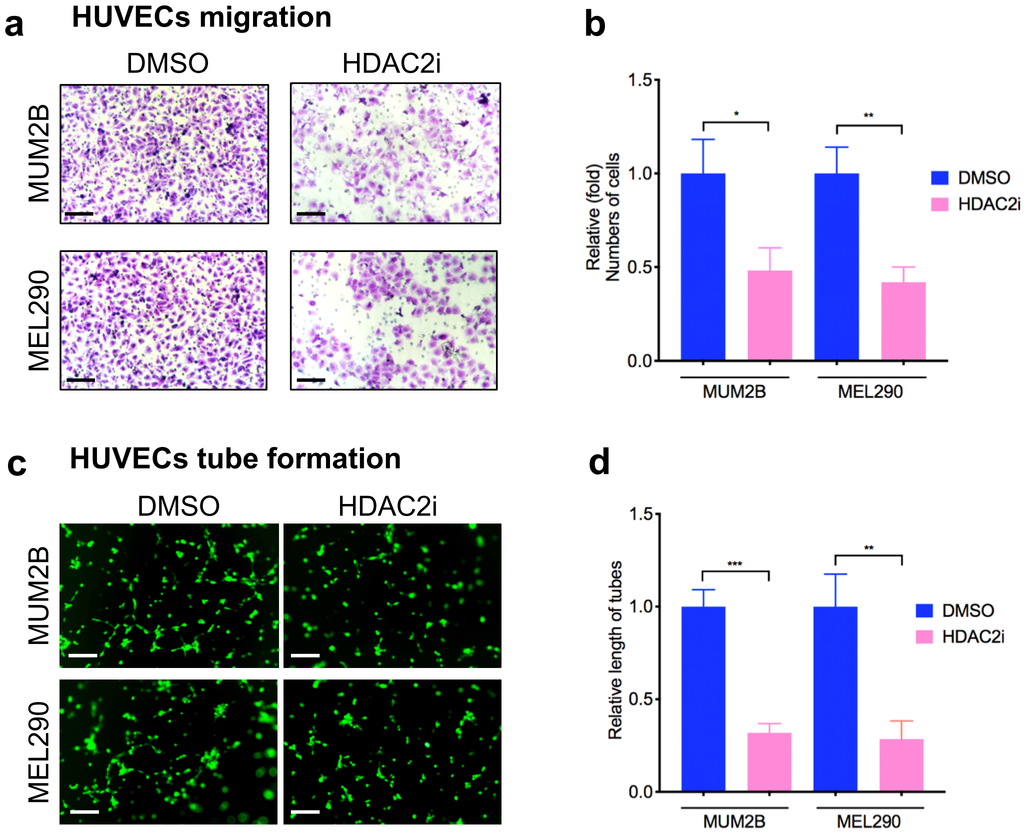

Supplementary Fig. 14. HDAC2 inhibition inhibits angiogenesis in uveal melanoma. a. HUVECs migration assay that investigates the effect of conditional medium from MUM2B and MEL290 cells treated with 50 µM HDAC2i for 24 h on the migration ability of HUVECs. Scale bars, 50 µm. **b**. Statistical analysis of the HUVECs migration assay. $n = 3$. Data are presented as means ± SD. Two-tailed unpaired Student's *t*-tests. **c**. HUVECs tube formation assay that investigates the effect of conditional medium from MUM2B and MEL290 cells treated with 50 µM HDAC2i for 24 h on HUVECs tubule formation. Scale bars, 50 µm. **d**. Statistical analysis of the HUVECs tube formation assay. $n = 3$. Data are presented as means ± SD. Two-tailed unpaired Student's *t*-tests.

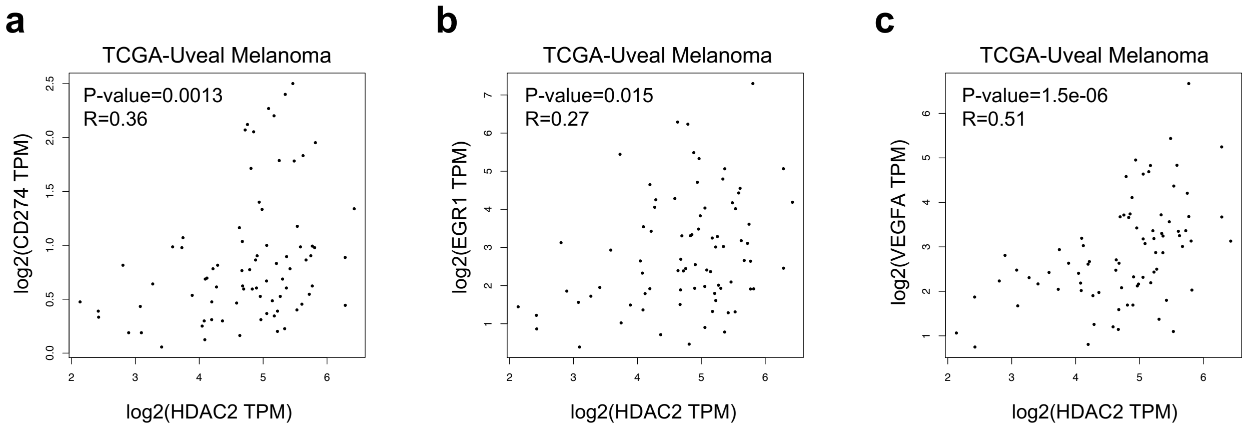

Supplementary Fig. 15. Correlation of HDAC2 with CD274, EGR1 and VEGFA in uveal melanoma from TCGA database. a-c Gene expression correlation analysis of HDAC2 and PD-L1 (**a**), HDAC2 and EGR1 (**b**), HDAC2 and VEGFA (**c**) in UM patients queried by TCGA database.

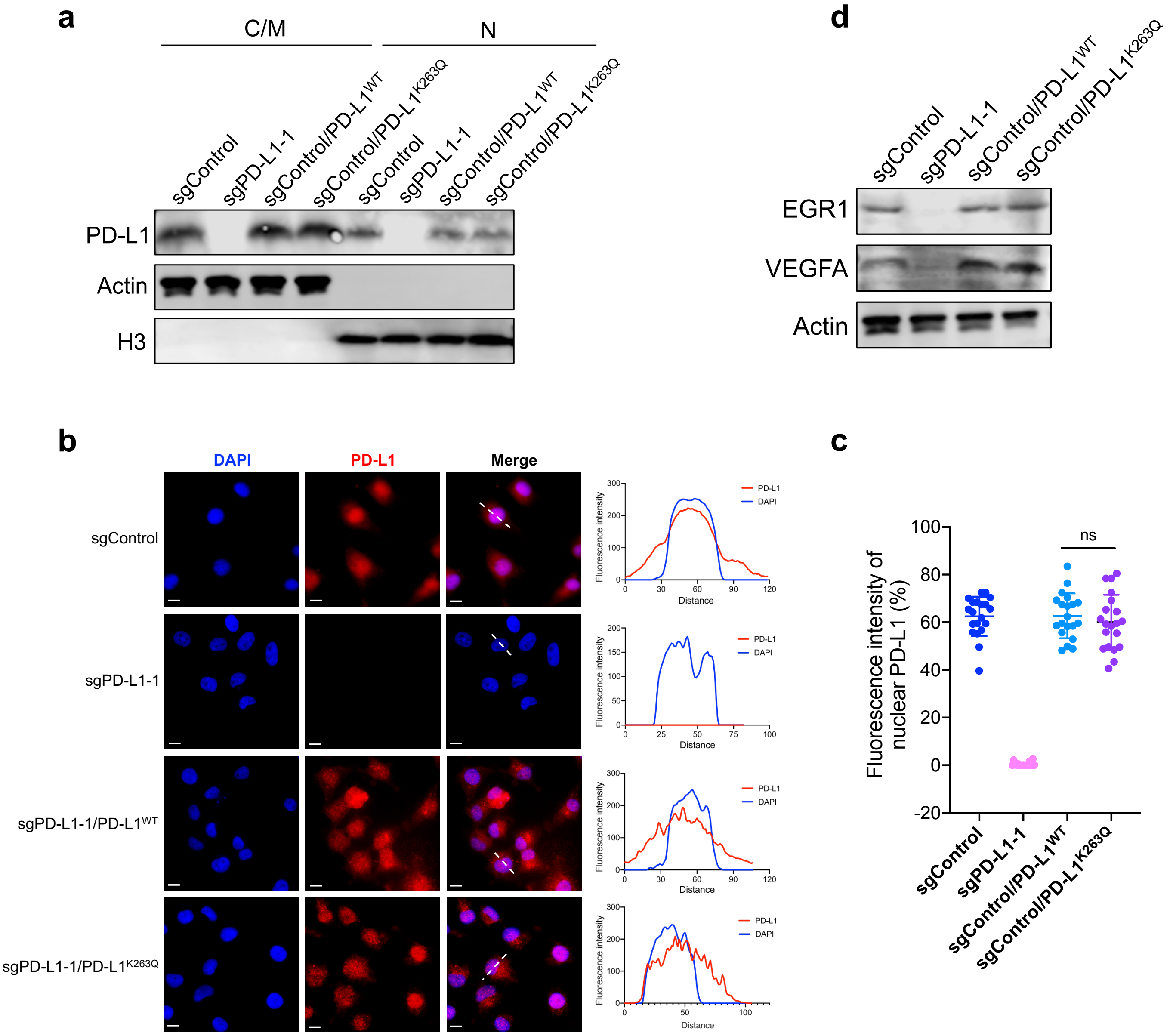

Supplementary Fig. 16. The effect of PD-L1K263Q on PD-L1 nuclear localization. **a** Western blot analysis of cytoplasmic/membrane (C/M) and nuclear (N) fractions of PD-L1 in MUM2B cells treated with the indicated constructs. **b** IF staining of PD-L1 in MUM2B cells treated with the indicated constructs. Left panel, representative IF staining images. Scale bars, 10 μm. Right panel, co-localization analysis of PD-L1 and DAPI by ImageJ software. **c** Statistical analysis of nPD-L1 fluorescence intensity (%) in MUM2B cells treated with the indicated constructs. n = 20. Data are presented as means ± SD. Two-tailed unpaired Student's t-tests. **d** Western blot images showing the protein levels of EGR1 and VEGFA in MUM2B cells treated with the indicated constructs.

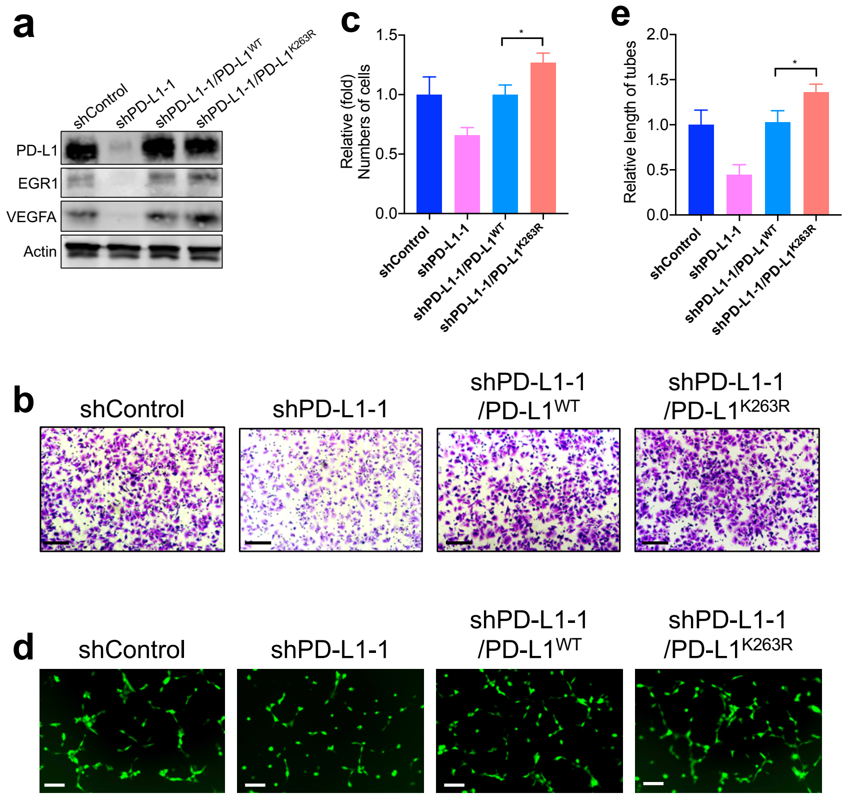

Supplementary Fig. 17. PD-L1 K263 deacetylation facilitates angiogenesis in breast cancer cells. **a** Western blot images showing the protein levels of PD-L1, EGR1 and VEGFA in MDA-MB-231 cells treated with the indicated constructs. **b** HUVEC migration assay that investigates the the effect of conditional medium from MDA-MB-231 cells treated with the indicated constructs on HUVECs migration ability. Scale bars, 50 µm. **c** Statistical analysis of the HUVEC migration assay. $n = 3$. Data are presented as means ± SD. Two-tailed unpaired Student's *t*-tests. **d** HUVEC tube formation assay that investigates the effect of conditional medium from MDA-MB-231 cells treated with the indicated constructs on HUVEC tubule formation ability. Scale bars, 25 µm. **e** Statistical analysis of the HUVEC tube formation assay. $n = 3$. Data are presented as means ± SD. Two-tailed unpaired Student's *t*-tests.

Supplementary Figs. 18-21. Unprocessed original scans of blots.

Fig. 1e

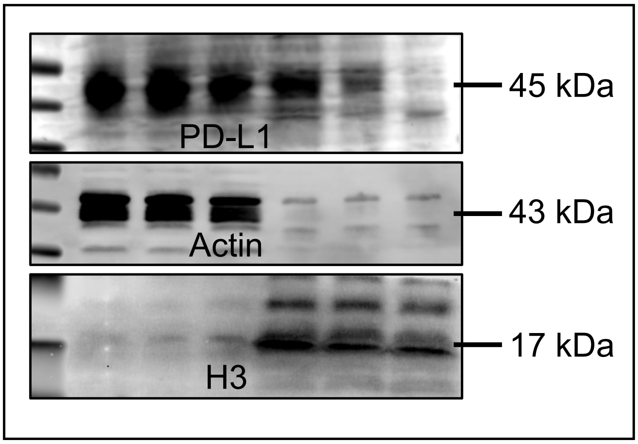

Fig. 2a

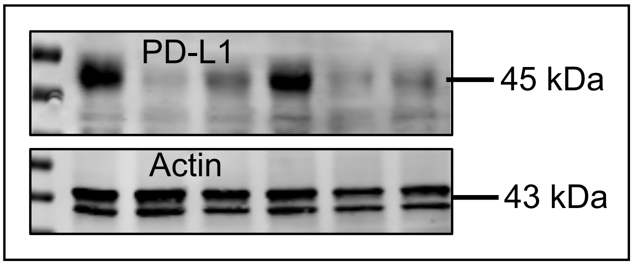

Fig. 3j

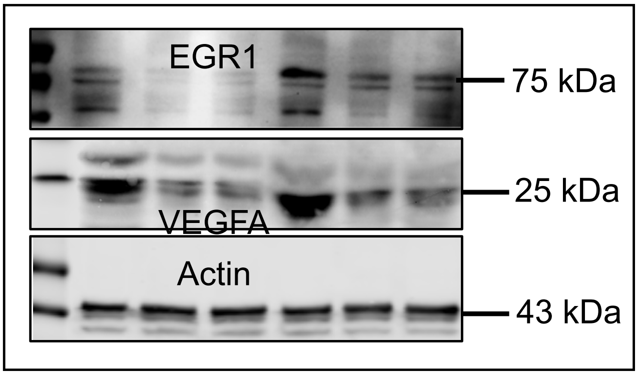

Fig. 5h

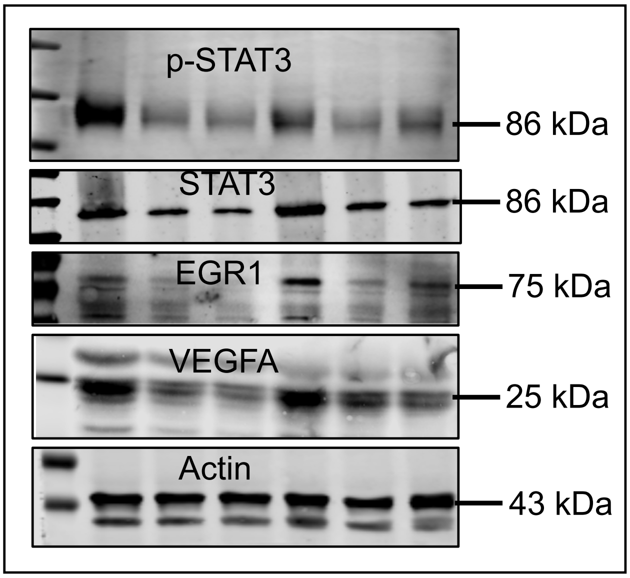

Fig. 6a

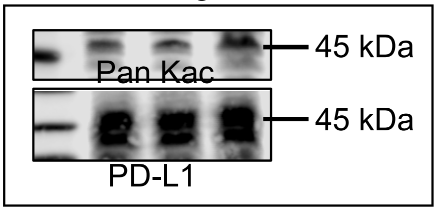

Fig. 6b

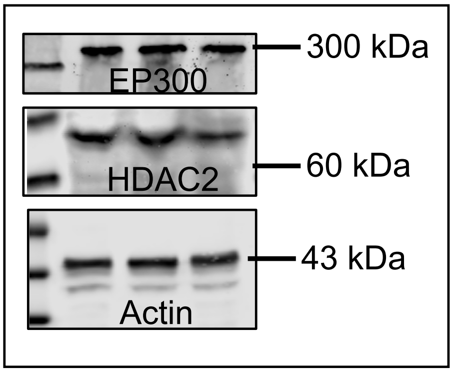

Fig. 6c

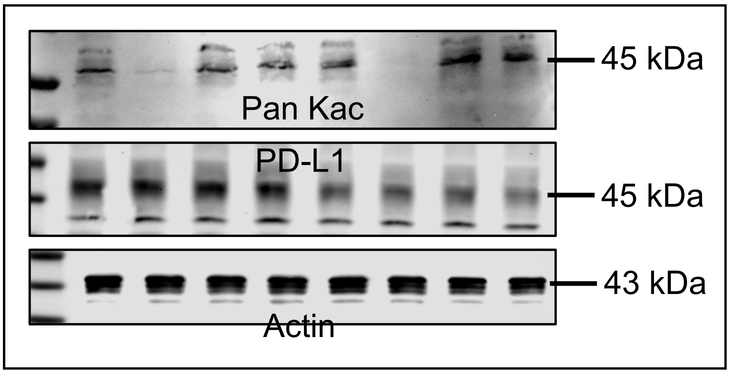

Fig. 6d

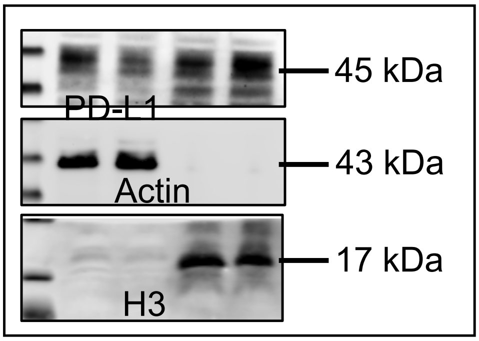

Fig. 6e

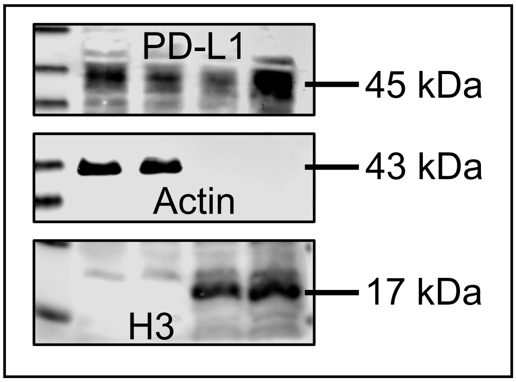

Fig. 6g

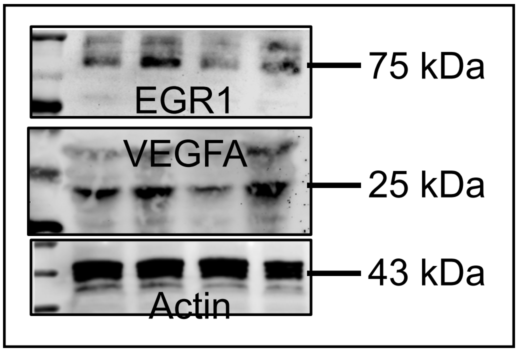

Fig. 6h

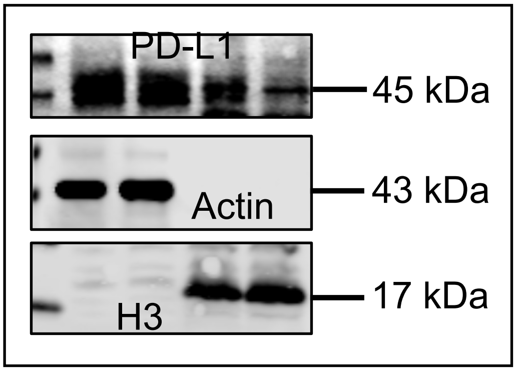

Fig. 6i

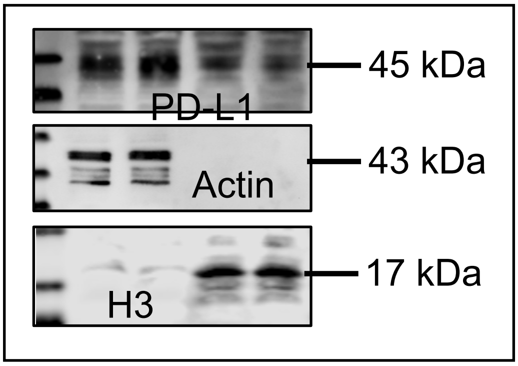

Fig. 6k

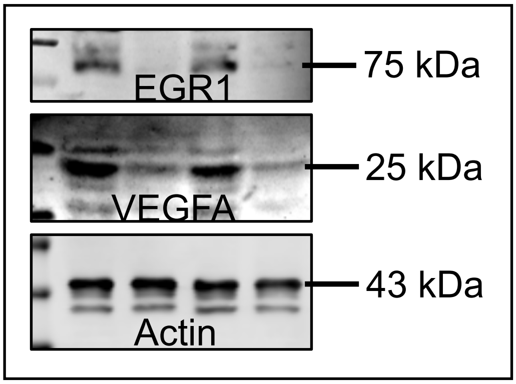

Fig. 6o

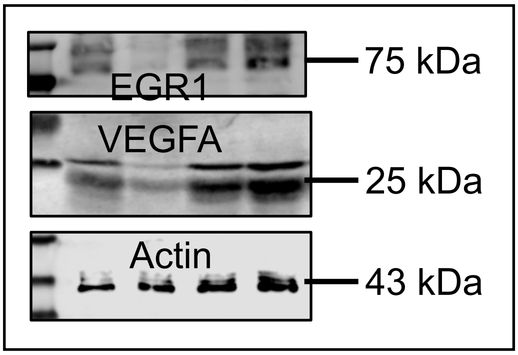

Fig. 6l

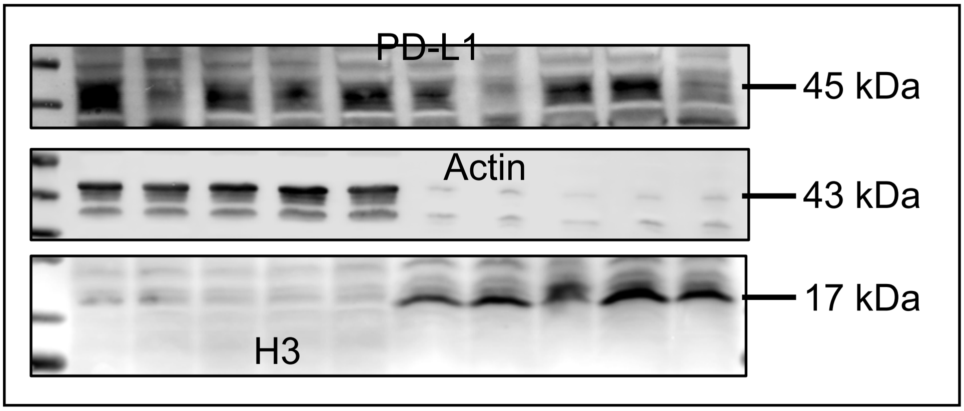

Fig. 8c

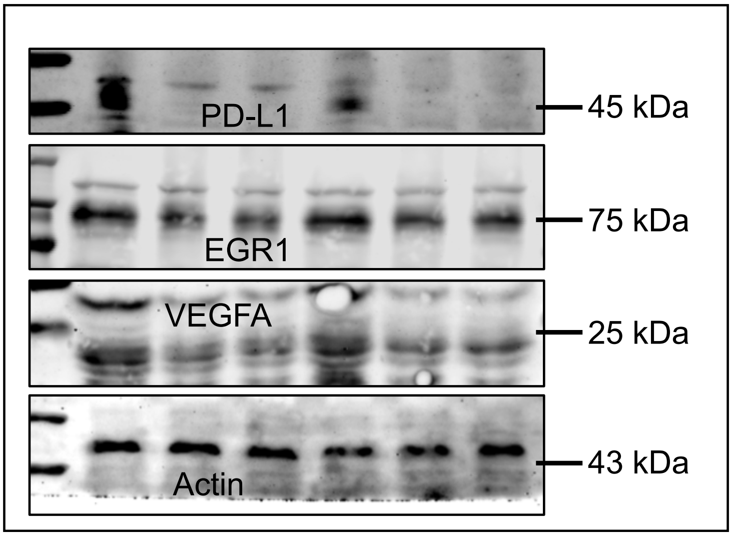

Fig. S4a

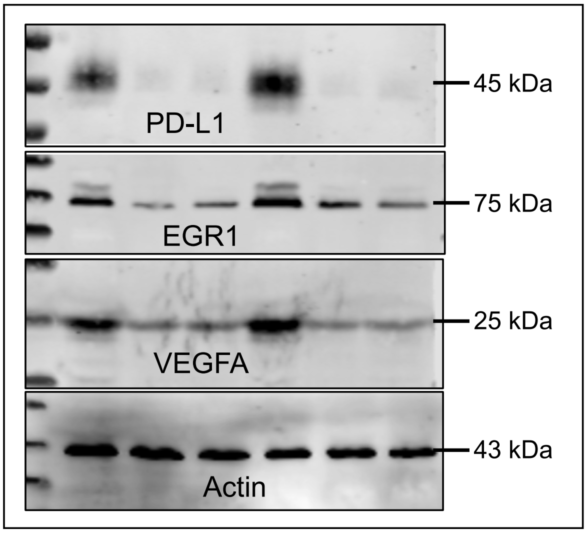

Fig. S1d

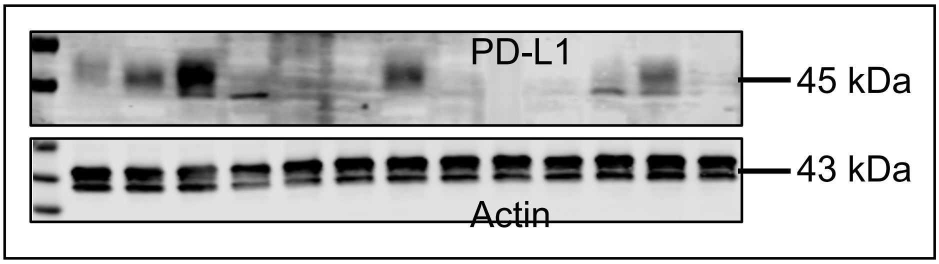

Fig. S1e

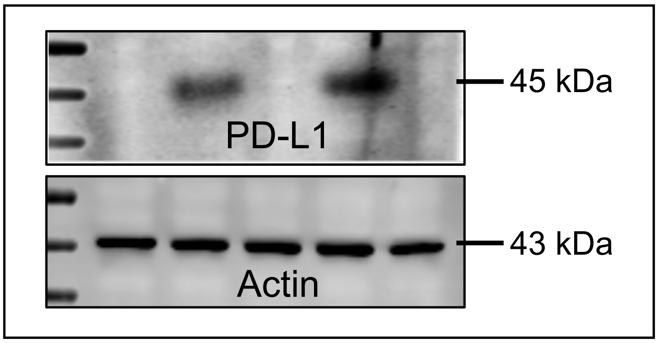

Fig. S1f

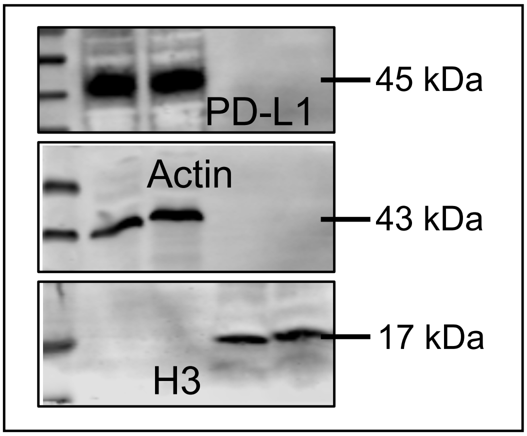

Fig. S6c

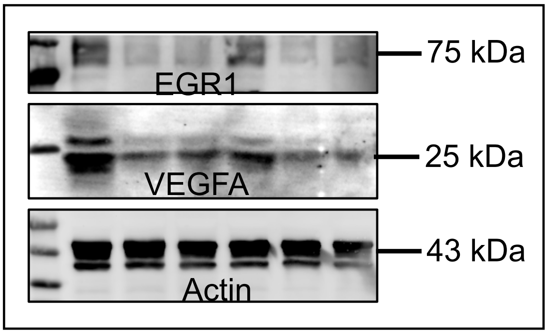

Fig. S7a

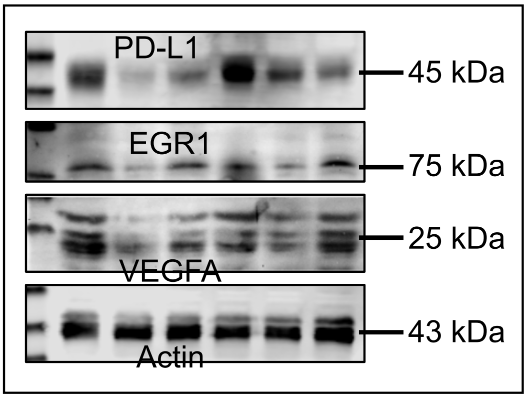

Fig. S8a

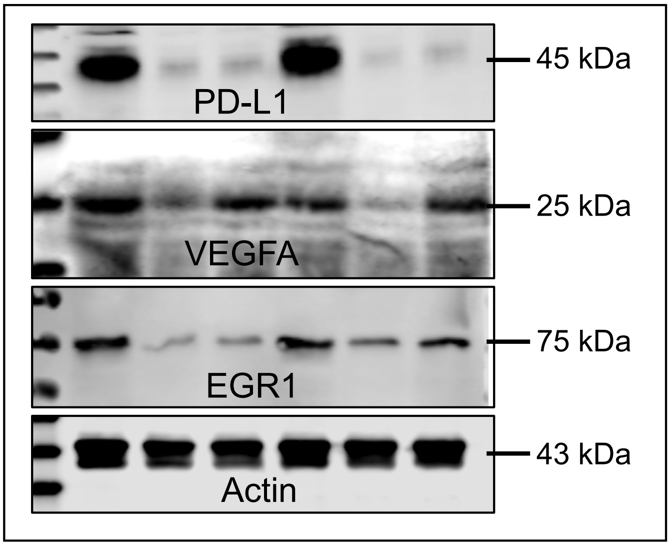

Fig. S10c

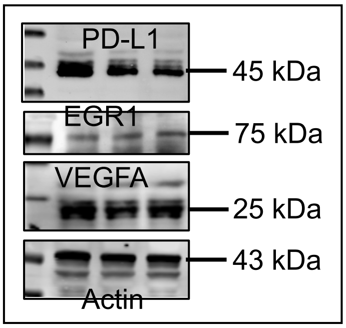

Fig. S16a

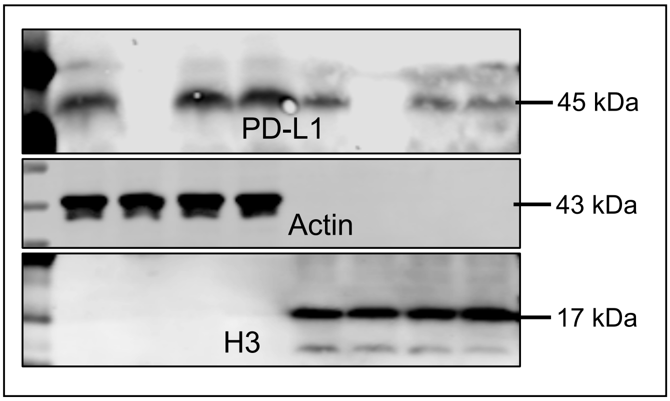

Fig. S16d

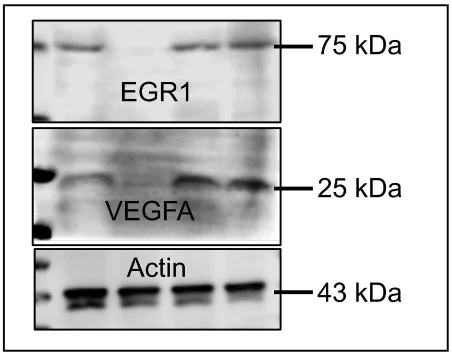

Fig. S17a

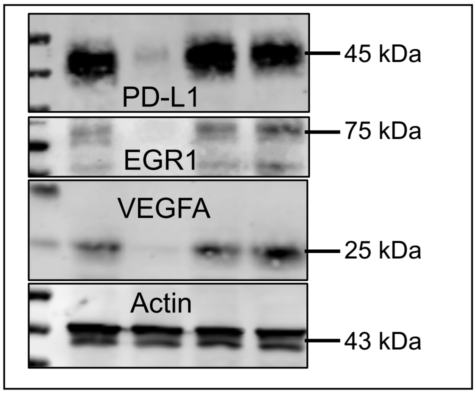

Table S1 Clinical characteristics and nuclear PD-L1 level in uveal melanoma patient cohorts in tissue chip

Patient No.	Sex (Female=0, Male=1)	Age	Time for recurrence (month, 0=No recurrence)	PD-L1 (Negative=0, Positive=1)	Nuclear PD-L1 proportion (%)
1	0	64	0	0	
2	1	67	22	1	45.27
3	1	52	8	1	66.93
4	1	37	0	0	
5	1	65	0	1	82.74
6	0	45	13	1	89.32
7	0	58	0	0	
8	1	32	32	1	66.49
9	1	21	36	1	7.49
10	0	66	20	1	52.76
11	0	69	0	0	
12	1	65	0	0	
13	1	23	13	1	48.62
14	1	23	0	1	4.18
15	0	52	37	1	6.92
16	1	80	0	0	
17	0	58	0	1	2.05
18	0	70	0	1	5.75
19	0	56	0	0	
20	1	68	0	0	
21	1	61	45	1	4.54
22	1	47	12	1	4
23	0	70	0	0	
24	0	52	0	0	
25	1	76	30	1	7.48
26	0	65	0	0	
27	1	46	0	0	
28	1	53	33	1	6.52
29	1	55	0	0	
30	1	48	27	1	14.52

Table S1 Clinical characteristics and nuclear PD-L1 level in conjunctival melanoma patient cohorts in tissue chip

Patient No.	Sex (Female=0, Male=1)	Age	PD-L1 (Negative=0, Positive=1)	Nuclear PD-L1 proportion (%)
1	1	59	0	
2	1	54	1	6.89
3	1	76	0	
4	1	46	0	
5	1	57	0	
6	1	57	0	
7	1	59	0	
8	1	59	0	
9	1	82	1	4.38
10	1	82	1	1.47
11	1	48	0	
12	0	29	0	
13	0	49	0	
14	0	49	1	2.48
15	1	35	0	
16	1	35	0	
17	1	47	0	
18	1	52	1	4.95
19	0	77	0	
20	0	42	0	
21	1	55	1	0.39
22	1	65	0	
23	1	31	0	
24	0	67	1	4.5
25	1	52	0	
26	1	73	0	
27	1	31	0	
28	1	78	0	
29	0	42	0	
30	1	73	0	
31	1	60	1	3.52
32	0	51	0	
33	1	52	1	1.47
34	1	73	0	
35	1	81	0	
36	1	47	0	
37	0	77	0	
38	1	54	1	3.75
39	1	63	0	
40	1	38	1	3.75
41	1	70	0	
42	1	18	0	

Table S1 Clinical characteristics and nuclear PD-L1 level in skin cutaneous melanoma patient cohorts in tissue chip

Patient No.	Sex (Female=0, Male=1)	Age	PD-L1 (Negative=0, Positive=1)	Nuclear PD-L1 proportion (%)
1	0	46	0	
2	1	60	0	
3	0	59	1	0.57
4	1	52	1	2.48
5	1	55	0	
6	0	38	0	
7	0	63	0	
8	0	41	1	3.18
9	1	52	0	
10	0	59	1	0.48
11	1	61	1	5.92
12	1	71	0	
13	1	51	0	
14	1	45	1	0.49
15	1	37	0	
16	1	38	0	
17	1	25	0	
18	1	65	1	0.19
19	1	38	0	
20	0	32	0	
21	0	45	1	0.42
22	1	40	1	0.85
23	0	77	1	2.48
24	1	49	0	
25	1	41	1	7.59
26	1	51	1	1.48
27	0	46	0	
28	1	61	0	
29	1	72	0	
30	1	49	1	4.28
31	1	53	0	
32	1	80	0	
33	0	42	1	2.49
34	0	88	0	
35	0	58	0	

Table S2. Primers used in this study

RT-qPCR primers:

Gene	Sequences (5’-3’)
ACTB-Forward	TGGCACCCAGCACAATGAA
ACTB-Reverse	CTAAGTCATAGTCCGCCTAGAAGCA
CD274-Forward	AAACAATTAGACCTGGCTG
CD274-Reverse	TCTTACCACTCAGGACTTG
PER1-Forward	ACCTGACCGCAGAGTCTTTT
PER1-Reverse	CAAGGTGTTGCCACTGTTGG
VEGFA-Forward	AGGAGGAGGGCAGAATCATCA
VEGFA-Reverse	CTCGATTGGATGGCAGTAGCT
STAT3-Forward	CAGCAGCTTGACACACGGTA
STAT3-Reverse	AAACACCAAAGTGGCATGTGA

ChIP-qPCR primers:

Gene	Sequences (5’-3’)
EGR1-site a-Forward	CCTACCAGGGAGTGGCAAAA
EGR1-site a-Reverse	ACTCCCACTGTCTACCGAGT
EGR1-site b-Forward	ACACCCTCCCCCACCTAATC
EGR1-site b-Reverse	GTGCTTGACGACACCGAGAG
EGR1-site c-Forward	TAGGGAAGCCCCTCTTTCGG
EGR1-site c-Reverse	TCCTTGTGGTGAGGGGTCA
EGR1-site d-Forward	GCGACCCGGAAATGCCATATAA
EGR1-site d-Reverse	GGTGCTGCCCAAATAAGGGT
EGR1-site e-Forward	GAAGGACCGTGATCCTTGGC
EGR1-site e-Reverse	ACAGCTGACGCAAATACGGA
EGR1-site f-Forward	CTCGTCCTCCAGTGATTGCTT
EGR1-site f-Reverse	TGGTTTGGCTGGGGTAACTG
